# Two-dimensional nanomaterials induced nano-bio interfacial effects and biomedical applications in cancer treatment

**DOI:** 10.1186/s12951-024-02319-5

**Published:** 2024-02-18

**Authors:** Yan Wang, Xiao Zhang, Hua Yue

**Affiliations:** 1 State Key Laboratory of Biochemical Engineering, Institute of Process Engineering, Chinese Academy of Sciences, Beijing, 100190 China; 2 Key Laboratory of Biopharmaceutical Preparation and Delivery, Chinese Academy of Sciences, Beijing, 100190 China; 3 School of Chemical Engineering, University of Chinese Academy of Sciences, Beijing, 100049 China

**Keywords:** Two-dimensional nanomaterials, Nano-bio interfacial effects, Cancer treatment

## Abstract

Two-dimensional nanomaterials (2D NMs), characterized by a large number of atoms or molecules arranged in one dimension (typically thickness) while having tiny dimensions in the other two dimensions, have emerged as a pivotal class of materials with unique properties. Their flat and sheet-like structure imparts distinctive physical, chemical, and electronic attributes, which offers several advantages in biomedical applications, including enhanced surface area for efficient drug loading, surface-exposed atoms allowing precise chemical modifications, and the ability to form hierarchical multilayer structures for synergistic functionality. Exploring their nano-bio interfacial interactions with biological components holds significant importance in comprehensively and systematically guiding safe applications. However, the current lack of in-depth analysis and comprehensive understanding of interfacial effects on cancer treatment motivates our ongoing efforts in this field. This study provides a comprehensive survey of recent advances in utilizing 2D NMs for cancer treatment. It offers insights into the structural characteristics, synthesis methods, and surface modifications of diverse 2D NMs. The investigation further delves into the formation of nano-bio interfaces during their in vivo utilization. Notably, the study discusses a wide array of biomedical applications in cancer treatment. With their potential to revolutionize therapeutic strategies and outcomes, 2D NMs are poised at the forefront of cancer treatment, holding the promise of transformative advancements.

**Graphical Abstract:**

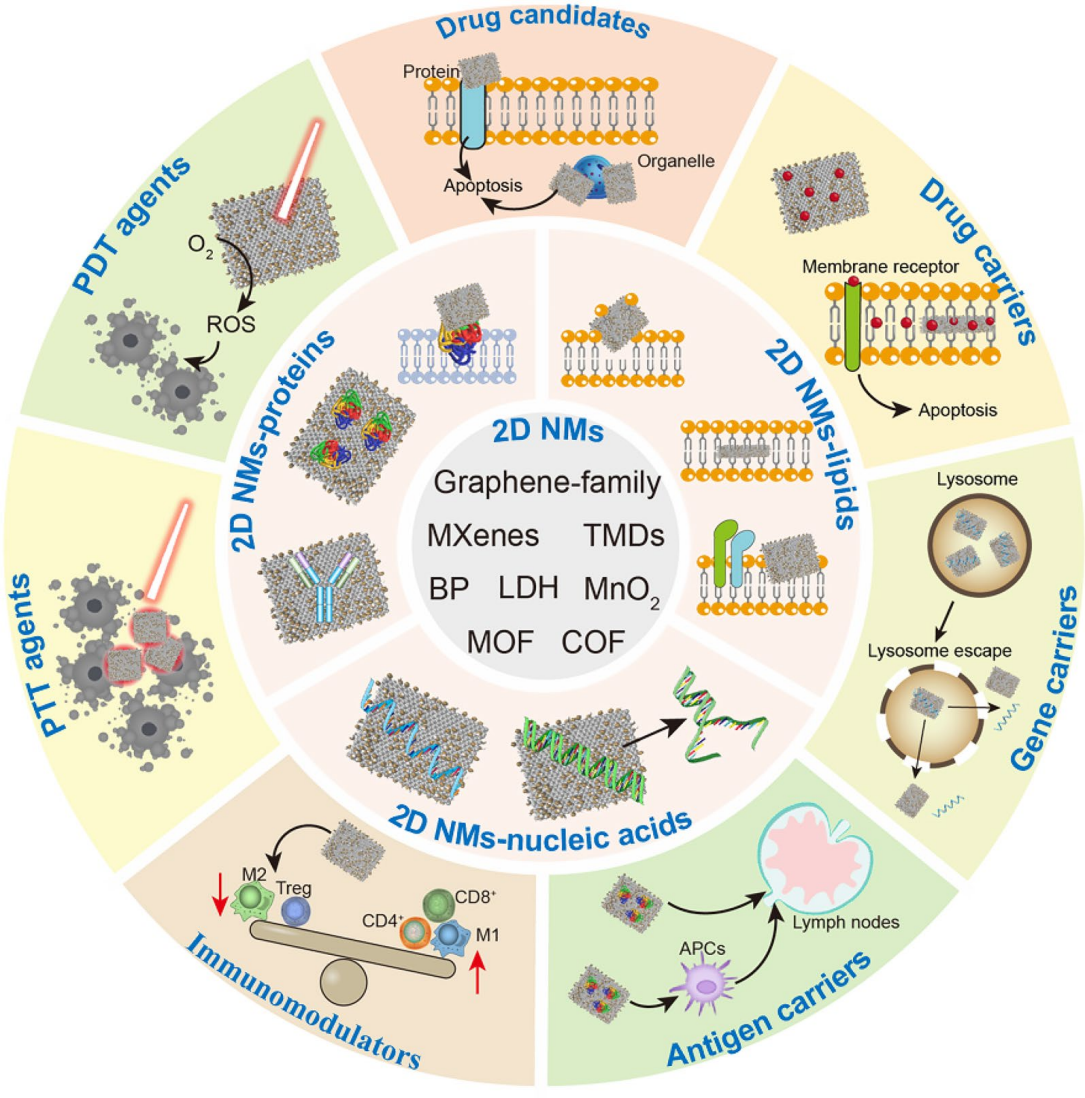

## Introduction

Two-dimensional nanomaterials (2D NMs) constitute a class of nanomaterials with a large number of atoms or molecules arranged in one dimension (typically thickness), while having tiny dimensions in the other two dimensions [1–4]. These materials often exhibit unique physical, chemical, and electronic properties due to their distinctive structure, finding significant applications in various fields such as nanotechnology, electronics, materials science, and biomedical research [5–8]. Currently developed 2D NMs encompass a diverse range of materials, including graphene-family nanomaterials, transition metal carbides and/or nitrides (MXenes), transition metal dichalcogenides (TMDs), black phosphorus (BP), layered double hydroxide (LDH), metal–organic framework (MOF) and covalent-organic framework (COF), manganese dioxide (MnO_2_), among others [9–14].

In comparison to nanomaterials of other dimensions, 2D NMs exhibit distinctive structural characteristics [15]. (1) Enhanced surface area: 2D NMs possess a significantly larger surface area, facilitating efficient drug loading and enabling highly integrated therapeutic effects. (2) Surface-exposed atoms: The majority of atoms constituting 2D NMs are exposed on their surface, dictating unique physicochemical properties and allowing for precise chemical modifications. (3) Hierarchical multilayer structures: 2D NMs can form hierarchical multilayer structures, accommodating the insertion of various molecules. This feature endows them with synergistic coupling effects and cooperative functionalities.

The structural characteristics of the aforementioned 2D NMs dictate unique nano-bio interfacial effects with biological components, mediating corresponding physiological functions and guiding their rational applications in the field of biomedicine. Particularly, these nano-bio interfacial effects play a pivotal role in the context of the global health challenge—cancer. The interaction between 2D NMs and cancer cells can lead to enhanced cellular uptake and targeted drug delivery, improving the efficacy of anticancer treatments. Additionally, the modulation of immune responses and the potential for combining therapeutic agents within the 2D NMs structure contribute to the development of innovative cancer treatment strategies. Existing synopses have contributed valuable insights into the theoretical frameworks and practical applications of certain 2D NMs, focusing on nano-bio interactions, drug loading, and bioactivity [16–19]. However, an in-depth analysis encompassing a diverse array of 2D NMs' nano-bio interfacial effects, mechanisms, and rational design specifically for cancer treatment remains lacking in the current literature.

**Scheme 1:**
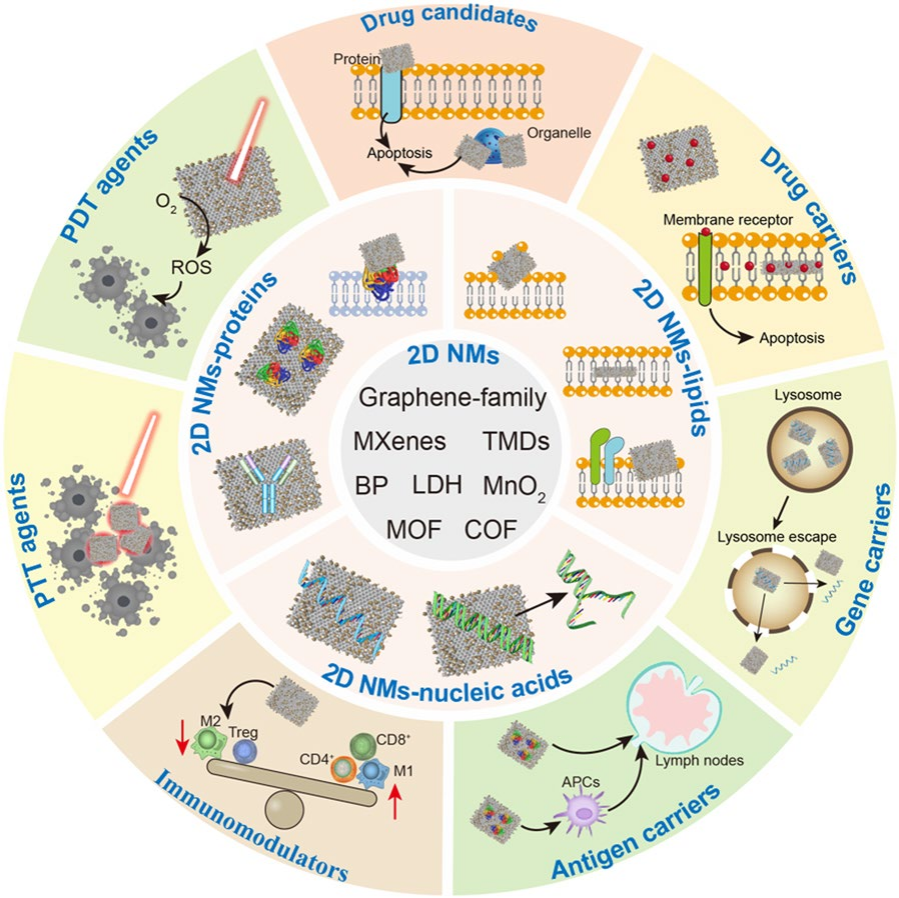
Schematic illustration of nano-bio interfacial effects of 2D NMs and their applications in cancer treatment

To tackle this gap, we present a comprehensive survey of recent breakthroughs in the cancer treatment of distinctive 2D NMs owing to their nano-bio interfacial effects (Scheme 1). Primarily, we offer an intricate examination of the structural attributes, synthesis techniques, and surface modifications of diverse 2D NMs. Subsequently, we provide an encompassing analysis of their nano-bio interfaces which are frequently formed during their utility in vivo. Finally, we discuss their detailed applications in the field of cancer treatment, including 2D NMs as drug candidates, chemo/gene-therapeutic drug carriers, antigen carriers, immunomodulators, photothermal therapy (PTT) agents and photodynamic therapy (PDT) agents.

## Characteristics, preparations and surface modifications of 2D NMs

### Graphene-family nanomaterials

K. Novoselov and A. Geim's pioneering work in 2004, extracting graphene from graphite, introduced the graphene family of nanomaterials into biomedical science. Graphene, a prominent 2D NM, exhibits a hexagonal lattice of sp^2^-bonded carbon atoms, with a mere thickness of 0.35 nm compared to previous dozen nanometers [20]. Serving as the foundation for various carbon-based nanomaterials, including fullerenes and graphite, graphene-family nanomaterials display remarkable thermal, mechanical, and electronic properties vital for biomedical applications. For instance, graphene showcases exceptional thermal conductivity (~ 5000 w/(MK)), electric conductivity (~ 15,000 cm^−2^/(VS)), a high specific surface area (2630 m^2^/g), impressive transparency to visible light (~ 97.7%), and a Young’s modulus (~ 1.0 TPa) [20–22]. However, this family encompasses diverse materials like pristine graphene, graphene oxide (GO), and reduced GO (rGO), each offering distinct features that impact their applications in biomedicine.

Pristine graphene, with its hydrophobic nature and strong inter-sheet interactions, poses challenges in liquid dispersion due to aggregation. Conversely, GO's surface, rich in oxygen functional groups like hydroxyl, epoxy, and carboxyl groups [23], significantly enhances its solubility and biocompatibility. Despite these advantages, GO tends to aggregate in certain solutions, reducing its efficiency in delivering bioactive substances and exhibiting toxicity in vivo, hindering its clinical translation. To address these issues, modifying GO's surface with functional groups improves its dispersibility and biocompatibility. Biopolymers such as polyethylene glycol (PEG) poly (acrylic acid) (PAA), poly lysine (PLL), chitosan oligosaccharide (CS) have been utilized to enhance the biocompatibility and dispersibility of graphene-family NMs [24–27]. For instance, PEGylation of nanosized GO (NGO-PEG) has been employed for hydrophobic drug delivery [28]. Reduction processes significantly decrease the oxygen content of rGO, altering its hydrophobicity, and surface modification with hydrophilic polymers further enhances its biocompatibility. Thus, the distinct physical and chemical properties of graphene-family NMs impact their utility in biomedical applications, such as drug delivery, biosensing, and tissue engineering, potentially revolutionizing biomedical technology [29].

### MXenes

MXenes, a novel class of 2D materials discovered by Yury Gogotsi and Michel W. Barsoum in 2011, comprise transition metal carbides, nitrides, and carbonitrides derived from MAX phases [30]. These MAX phases are layered ternary compounds with the formula M_n+1_AX_n_ (n = 1–3), where M represents transition metals like Ti, Zr, V, and A denotes elements from groups 13 to 14 [31–33]. MXenes, with formulations M_2_X, M_3_X_2_, and M_4_X_3_, possess tunable thicknesses impacting their physicochemical properties, blending ceramic and metallic behaviors. These materials offer unique properties for biomedical applications. Like other 2D materials, MXenes exhibit a high specific surface area conducive for functionalization and modification. From a ceramic perspective, they showcase high hardness and corrosion resistance. Moreover, certain MXenes like Ti_3_C_2_, Nb_2_C, and MoC_2_ boast high thermal conductivity, excellent photothermal conversion, and strong near-infrared (NIR) absorption) [34–36]. Some MXenes integrated with metals like Ta and W demonstrate robust X-ray attenuation, while manganese-incorporated MXenes exhibit magnetic properties [37, 38].

Synthetic methods for MXenes, either top-down or bottom-up, influence their structure and properties [39]. Top-down methods involve chemical etching and delamination, while bottom-up approaches, such as chemical vapor deposition (CVD), offer atomic-level control over their synthesis [40]. Despite their advantages for biomedicine, 2D MXenes face challenges such as aggregation in physiological environments. To address this, surface modifications, like coatings with biocompatible compounds or polymers (e.g., PEG, soybean phospholipid, PVA, PVP), are crucial [38, 41–43]. For example, Lin et al. reported enhanced biocompatibility and effective tumor eradication using SP-modified Ti_3_C_2_ nanosheets (Ti_3_C_2_-SP) under 808 nm NIR laser irradiation. These modifications enhance stability, paving the way for potential biomedical applications of 2D MXenes [44].

### TMDs

TMDs consist of transition metals (e.g., Mo, W) and chalcogens (S, Se, Te) in a 1:2 ratio, forming atomic layers with a sandwich-like X-M-X structure bound by van der Waals forces [45, 46]. Their intriguing structure and remarkable optical and physicochemical properties have sparked substantial interest in recent years. TMDs boast unique characteristics pertinent to biomedical applications. Firstly, their surface lacks dangling bonds, rendering 2D TMDs highly stable in liquid and air [47]. Secondly, the combination of chalcogen and transition-metal atoms endows TMDs with distinct optical properties. Additionally, TMDs, thinner and more flexible than other 2D NMs, exhibit lower cytotoxicity compared to materials like graphene [12].

Various synthesis methods, such as mechanical and liquid/gas-phase exfoliation, and CVD, have been employed for TMD production [48]. While mechanical exfoliation is convenient, it damages the morphology of TMDs, limiting efficiency. Liquid/gas-phase exfoliation methods, particularly ultrasound-mediated Li-intercalation, offer efficient production and control over TMDs' layer numbers [49, 50]. For instance, ceMoS_2_ obtained via Li-intercalation exhibited verified optical and photothermal properties [51]. CVD stands out as a practical approach for producing large quantities and high-quality TMDs with adjustable shape and thickness by tweaking parameters like growth temperature and gas flow [52, 53].

However, the hydrophobic nature of TMDs restricts their biomedical applications, necessitating modifications for enhanced hydrophilicity, improved biodistribution, and prolonged circulation in the bloodstream. Various polymers, including chitosan, PAA, PEG, polyethyleneimine (PEI), PEG-PEI, amphiphilic copolymers, and block copolymers, serve as functional materials for surface modifications to impart hydrophilic properties to TMDs, broadening their potential biomedical utility [54–62].

### BP

BP, the most stable phosphorus allotrope, exhibits distinctive properties vital in biomedical contexts, distinguished by its orthogonal crystal structure [63]. Firstly, its puckered lattice configuration imparts a remarkably high specific surface area, enhancing its potential in various applications [64]. Secondly, BP showcases layer-dependent light absorption across ultraviolet and infrared regions, making it an excellent photosensitizer with wide band gaps from 0.3 eV to 2.0 eV. Additionally, BP demonstrates exceptional photothermal conversion efficiency at specific wavelengths (e.g., 808 nm and 1064 nm), offering promise in photothermal therapies [13, 65]. Moreover, BP exhibit biodegradability, renal clearance, and commendable biocompatibility, crucial for biomedical applications.

Synthesis methods for BP nanosheets involve top-down approaches like mechanical exfoliation and bottom-up techniques such as CVD or wet-chemical methods [66]. Mechanical exfoliation remains a prevalent method for obtaining BP nanosheets, producing single-layer nanosheets of a mere 0.85 nm thickness [66]. Other methods like liquid-phase stripping, CVD, and wet-chemical routes have also been adopted for BP nanosheet synthesis.

However, BP nanosheets are prone to oxidation and degradation in aqueous media, leading to the formation of phosphates and phosphonates [67]. While this degradation reduces toxicity, it concurrently compromises BP's photothermal performance, limiting its biomedical utility [68]. To address these challenges, researchers have been exploring surface modification methods. Strategies involving π − π stacking interactions with aromatic 1-pyrenylbutyric acid have demonstrated enhanced stability and biological activity of BP for targeted tumor therapy [69]. Additionally, coatings with polydopamine (PDA) and PEG-PEI have proven effective in improving BP stability [70, 71]. These surface modifications not only enhance stability but also offer increased active sites for diverse applications due to BP's inert surface lacking reactive functional groups.

### LDH

LDH is characterized by a general formula [M_1-x_^2+^M_x_^3+^(OH)_2_]^x+^[A_x/n_]^n−^·mH_2_O [72, 73], holds promise for biomedical applications due to their unique attributes. Firstly, their interlayer structure allows efficient loading of biologically active molecules (like genes, proteins) and drugs, demonstrating potential for targeted therapy [74]. Secondly, LDH nanosheets exhibit pH-responsive release in acidic environments, aiding targeted therapy while displaying high biocompatibility and degradability [75]. Thirdly, LDH's intercalation chemistry and rare earth elements enable medical imaging applications, serving as contrast agents (CAs) for CT imaging or presenting excellent T_1_-magnetic resonance imaging (MRI) performance for cancer diagnosis and treatment [76].

Various preparation methods, including co-precipitation, anionic exchange, exfoliation-reconstruction, and hydrothermal techniques, form the basis for LDH nanosheets' biomedical applications. Co-precipitation, the simplest method, adjusts pH to obtain LDH, while anionic exchange introduces different anions into LDH interlayers [77]. The recently developed exfoliation-reconstruction technique and high-temperature hydrothermal methods are also employed.

Aggregation of nanomaterials in the body diminishes their biomedical efficacy. LDH’s ability to absorb compounds with opposite charges results in aggregation, hampering cellular endocytosis. To address this challenge, surface modification of LDH nanosheets with bioactive compounds like PEG, silica, and BSA enhances stability, extends circulation time in the bloodstream, and safeguards their properties for effective biomedical applications.

### MOF and COF

MOF and COF represent organic porous crystalline materials with distinct compositions; MOF is constructed with metal ions and organic linkers, while COF involves covalent bonds among lighter elements like C, O, N, and B [78, 79]. These frameworks have gained substantial attention owing to their intricate porous structures, providing extensive interaction not only on their surface but within their internal framework as well [80]. Their porous nature endows MOF and COF nanosheets with substantially larger specific surface areas and more active sites compared to other 2D NMs. Furthermore, their 2D counterparts exhibit improved dispersibility and stability compared to 3D structures [81]. The adaptability of their porous structure through chemical or physical methods enables customization for diverse applications.

Similar to other 2D NMs, the production of MOF and COF nanosheets can be accomplished via top-down and bottom-up approaches. Liquid ultrasonic exfoliation enables the extraction of 2D MOF and COF from their 3D structures [81]. Researchers have designed specific 2D COF by liquid sonication exfoliation, which possess biodegradability owing to the presence of hydrolysable boronate ester. Additionally, bottom-up fabrication methods involving soft-template-assisted techniques and surfactant modulation have been employed to produce 2D MOF and COF nanosheets [82, 83]. These methods allow the creation of various 2D M-TCPP(Fe) series, including Co, Cu, and Zn-based variants, emphasizing their versatility in synthesis [83].

While MOF and COF nanosheets demonstrate biocompatibility, their surface can be further tailored to enhance their efficacy. For instance, researchers have crafted ICG@COF-1 nanosheets through ultrasonic exfoliation and subsequently coated them with PDA, improving their stability and circulation time in the bloodstream [84]. These modifications play a crucial role in optimizing their utility in biomedical applications.

### MnO_2_

MnO_2_, displaying different crystallographic polymorphs like α-, β-, γ-, δ-, ε-, and λ- MnO_2_, exhibit diverse structures such as 1D, 2D, and 3D forms. The δ-MnO_2_ variant particularly displays a 2D layered structure. Recognized as layered metal oxides (LMOs), MnO_2_ nanosheets have garnered significant attention in biomedical applications due to their unique properties. Firstly, in the tumor microenvironment (TME), MnO_2_ nanosheets can degrade into Mn^2+^ ions upon exposure to H^+^ and glutathione (GSH), serving as contrast agents for T_1_-MRI [85, 86]. These nanosheets are biocompatible, as Mn^2+^ can be quickly eliminated by the kidneys, ensuring safety for clinical use. Additionally, they can decompose in tumor sites with excess H_2_O_2_, generating more oxygen, thereby alleviating tumor hypoxia. This characteristic enhances the effectiveness of PDT and radiation therapy and promotes the generation of singlet oxygen (^1^O_2_) for PDT via GSH response) [87–89].

MnO_2_ nanosheets, akin to other 2D NMs, can be obtained through top-down approaches like exfoliation methods or by in situ growth on other materials. Varied fabrication methods exist, such as the co-precipitation method used by Chen et al. to produce layered Na-MnO_2_ and subsequently exfoliate MnO_2_ from it by exchanging Na^+^ for H^+^ [90]. Novel wet-chemical and hydrothermal methods have also been proposed by Tang and Wang et al., respectively, to control the size and thickness of MnO_2_ nanosheets [85, 91].

While pristine MnO_2_ nanosheets possess low cost and non-toxic properties for a wide range of biomedical applications like PDT, PTT, and drug delivery, surface modification is crucial to enhance their performance. Tang et al. employed a sono-chemical method to modify MnO_2_ nanosheets with 2-S-(4-Isothiocyanatobenzyl)-1,4,7-triazacyclononane-1,4,7-triacetic acid modified BSA (BSA-NOTA) for improved stability and dispersion, specifically catering to positron emission tomography (PET) imaging requirements [91].

### Other 2D NMs

Besides the previously mentioned 2D NMs suitable for biomedical applications, there exist other promising candidates such as graphyne, graphitic carbon nitride (g-C_3_N_4_), boron nitride (BN), diverse metal nanosheets (e.g., Au, Pd, Pt, and Pt@Pd), and selenium-based compounds (e.g., InSe, Bi_2_Se_3_, Cu-Fe-Se, and Sb_2_Se_3_). 2D NMs exhibit exceptional physical and chemical traits owing to their unique sheet-like structures. However, existing 2D NMs often fall short of meeting the increasingly urgent demands in biomedicine. There is a need to explore novel 2D NMs possessing distinct properties specifically suited for clinical biomedical applications.

In summary, each type of 2D NM shares commonalities inherent due to special dimensional effects, while also exhibiting unique properties that set them apart. Graphene stands out with its hexagonal lattice structure, providing exceptional electronic properties. MXenes offer hydrophilicity and tunable surface chemistry derived from layered MAX phases. TMDs showcase semiconducting behavior and optoelectronic properties due to their layered structure. BP's anisotropic electronic structure and tunable bandgap result from its layered configuration. LDH possesses anion exchange capacity and biocompatibility with intercalated anions. MOFs and COFs feature high porosity and tunable chemical functionality in crystalline frameworks. MnO_2_, a transition metal oxide, displays excellent electrochemical activity in various polymorphs. These unique features make each 2D NM suitable for distinct applications, spanning electronics, optoelectronics, energy storage, and catalysis.

## Effects of nano-bio interfacial interactions by 2D NMs

When transported within the body, 2D NMs undergo a sequence of nano-bio interactions with various biological components, such as proteins, lipids, and nucleic acids, eliciting a range of interface effects. These interactions at the nano-bio interface play a pivotal role in shaping the biomedical applications as well as behavior and fate of these nanomaterials in biological systems [92, 93]. The intricate interplay between 2D NMs and these biological components underscores the significance of understanding nano-bio interactions to comprehend their behavior, biocompatibility, and potential applications within biological systems [16].

### 2D NMs-proteins interactions

**Fig. 1:**
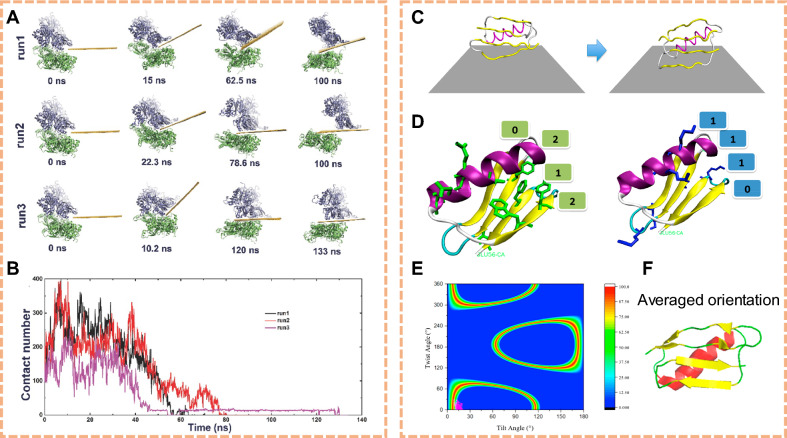
Effects of 2D NMs on protein stability. **A** Represent active intermediate structures during the inserting processes for all three replicas of independent trajectories, noted as traj1, traj2, and traj3. Dimer-I and dimer-II, taken from different strands, are colored with blue and green, respectively. **B** Time evolution of heavy atom contact number between two actin dimers. (Reproduced with permission from Ref. [^94^], Copyright 2016, John Wiley and Sons). **C** Initial and final snapshots of the double-point mutated protein GB1 on graphene from an example simulation. **D** Distribution of "planar" side-chain residues (left) and lysines (right) in β-sheet regions of protein GB1 that determines the adsorbed orientation of the protein GB1 mutant. **E** Heat map plot of possible orientation angle regions deduced using the SFG and ATR-FTIR measurements. Pink dots are the obtained orientations from the final 5 ns of MD simulation. **F** The averaged orientation (θ = 14°, ψ = 8°) of protein GB1 on graphene surface from the final 5 ns of simulation. (Reproduced with permission from Ref. [^95^], Copyright 2019, American Chemical Society)

2D NMs, such as graphene and TMDs, exhibit distinctive interactions with proteins that can profoundly influence protein conformation and stability. These nanomaterials can induce structural alterations in proteins, impacting their secondary structures, folding kinetics, and overall stability [17]. For example, Tian et al. used large-scale all-atom molecular dynamics simulations to reveal the interactions between GO nanosheets and actin filaments at molecular details (Fig. 1A) [94]. GO nanosheets can insert into the interstrand gap of actin tetramer (helical repeating unit of actin filament) and cause the separation of the tetramer which eventually leads to the disruption of actin filaments (Fig. 1B). Wei et al. chose protein GB1, an immunoglobulin G (IgG) antibody-binding domain of protein G, as a model protein to demonstrate how to preserve the protein native structure and control protein orientation after adsorbed onto the graphene surface (Fig. 1C) [95]. They found that such nano-bio interactions could destroy the α-helix structure of the wild-type protein GB1 on graphene, leading to the denaturation of the entire protein. However, a protein GB1 mutant with only two amino acids in the α-helical structure mutated could preserve the protein conformation and control its orientation on the graphene surface (Fig. 1D–F). Baimanov et al. found that human serum albumin, transferrin and fibrinogen underwent conformational changes like decrease in alpha helical content and increase in random coils upon interacting with MoS_2_ nanosheets, while immunoglobulins remained favorably stable [96]. These studies collectively emphasize the significant influence of 2D NMs on protein stability, providing valuable insights into their structural modifications and potential implications for biological function.

Actually, under physiological conditions, 2D NMs first interact with proteins in biological fluids and results in formation of protein corona, which significantly influences their functionality. The protein corona, formed upon the interaction of nanomaterials with proteins present in the biological milieu, alters their surface properties and biological identity. This corona can influence cellular uptake, biodistribution, and immune responses, thereby modulating the overall behavior and functionality of the nanomaterials in biological environments. Mo et al. showed that the size of BP can influence the identity and quantity of plasma proteins that form the corona, and in return, the corona could define the shape of ultra-small 2D NMs–corona complexes but does not change the shape of large 2D NMs [97]. Owing to the enrichment of immune proteins and other opsonins on the surface of BP nanomaterials, the uptake efficiency of BP–corona complexes by macrophages is higher, and the size-dependent cellular uptake pattern of BP–corona complexes is different from that of native nanomaterials.

Mitigating the influence of protein coronas on nanomaterials involves employing various strategies aimed at reducing or altering the protein-nanomaterial interactions. Surface modification and functionalization techniques serve as effective approaches to minimize protein adsorption onto nanomaterials. Introducing specific coatings or bio-compatible molecules, such as PEG or serum albumin, can deter protein binding, thus diminishing the formation of protein coronas. Precise control over nanomaterial properties, including size, shape, surface chemistry, and charge, offers another avenue to mitigate protein corona formation. Selecting or optimizing nanomaterial structures that exhibit reduced protein adsorption in biological environments helps alleviate the adverse effects of protein coronas on nanomaterial functionality.

Understanding the interplay between two-dimensional nanomaterials and protein structures, as well as the impact of protein coronas, is crucial for designing tailored nanomaterials with desired functionalities for various biomedical applications. This comprehension facilitates the development of nanomaterials with enhanced biocompatibility, improved targeting efficiency, and minimized adverse effects, contributing to their utility in drug delivery, diagnostics, and therapeutic interventions. However, continued research is imperative to unravel the complexities of these interactions and exploit them effectively in the design and application of nanomaterials in biomedicine.

### 2D NMs-lipids interactions

**Fig. 2:**
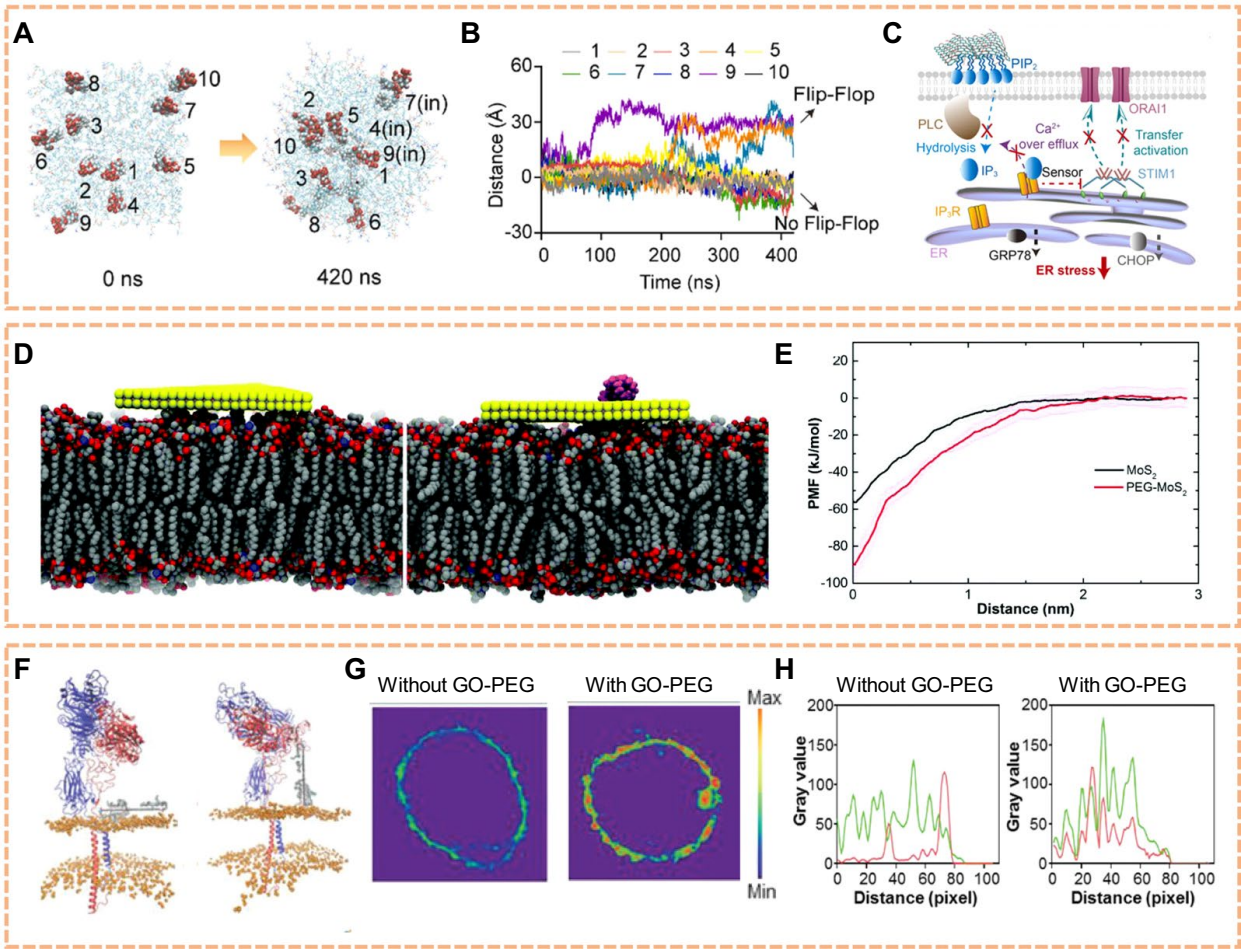
Effects of 2D NMs on lipid disturbance. **A** Detailed numbering for each PIP_2_ molecule at the start of the simulations (0 ns), and their terminal orientations (420 ns). **B** Vertical distance between the PH-domain hydrolysis site of each PIP_2_ molecule and the inner membrane surface. The upward elongation trend of #4, #7 and #9 PIP_2_ supports the frequent “flip-flop switching”, leading to more hydrolysis sites embedded within membrane. **C** Diagram of P-sheet regulated IP3 signal transduction to inhibit ER stress mechanism. (Reproduced with permission from Ref. [^99^], Copyright 2022, Elsevier). **D** Final snapshots of the large MoS2 and MoS2-PEG nanoflakes binding to the macrophage membrane surface. **E** Potential of mean force (PMF) curve showing the free energy as the MoS2/MoS2-PEG moving along the z-direction, normal to the macrophage membrane surface. (Reproduced with permission from Ref. [^100^], Copyright 2019, Royal Society of Chemistry). **F** Structure at the initial moment of production simulation with GO-PEG in the horizontal mode and vertical mode. **G** Representative fluorescence imaging on lipid aggregation of macrophage with and without GO-PEG stimulation. The membrane lipids were labeled with lipophilic β-BODIPY dye. **H** Typical linear overlay analysis of zoomed section in white rectangular region suggests that integrin β8 and talin-1 co-localization increases after GO-PEG stimulation. (Reproduced with permission from Ref. [^102^], Copyright 2021, John Wiley and Sons)

The interaction between 2D NMs and cellular membranes has been shown to intricately influence the composition of phospholipids within the membrane bilayer. Upon exposure to various two-dimensional nanomaterials, alterations in the lipid composition of cellular membranes have been observed. These nanomaterials often induce modifications in the distribution and arrangement of phospholipid species within the membrane. The introduction of nanomaterials may lead to changes in the lipid packing density, fluidity, and phase behavior of the membrane, impacting its structural integrity and functionality. Tu et al. showed experimentally and theoretically that Graphene nanosheets can penetrate into and extract large amounts of phospholipids from the cell membranes because of the strong dispersion interactions between graphene and lipid molecules [98]. Huang et al. found that PEGylated graphene oxide could cause a flip-flop orientation shift of phosphatidylinositol-4,5-bisphosphate lipid within the membrane, which restricted the hydrolysis site to prevent cleavage-mediated inositol 1,4,5-trisphosphate (Fig. 2A, B) [99]. This nano-bio interfacial effect reduced Ca^2+^-related endoplasmic reticulum stress to protect the neurons of Parkinson's disease mice (Fig. 2C). Gu et al. performed molecular dynamics simulations to examine the atomic detailed interactions of MoS_2_ and MoS_2_-PEG nanoflakes with a realistic model of the macrophage membrane (Fig. 2D) [100]. The characteristics of slower/prolonged membrane penetration and stronger membrane adsorption of MoS_2_-PEG compared to pristine MoS_2_ explain why it triggers more sustained stimulation and higher cytokine secretion in macrophages (Fig. 2E). In summary, the interaction of 2D NMs with cellular membranes can alter phospholipid composition, impacting membrane structure and function.

Furthermore, 2D NMs can mediate the formation of nanopores or nanochannels due to their high surface activity and tunable physical/chemical properties. When these materials interact with biological or artificial membranes, their surfaces can induce membrane restructuring or assembly. This process can lead to the creation of pores by serving as templates or catalysts through the formation of microdefects, fissures, or gaps on the surface. The nanopores formed can be utilized in molecular sieving, drug delivery, biosensing, and other applications in nanotechnology. For example, Chen et al. experimentally evidenced the sandwiched graphene–cell membrane superstructure and theoretically demonstrated that the transport of such a 2D nanosheet integrated inside the cell membrane exhibited the transition of diffusion patterns from Brownian to Lévy and even directional dynamics, modulated by its interaction with the membrane interior [101]. To gain insights into such an intriguing transition, the details of the superstructures were examined and first identify the sandwiched GO–induced pore in the leaflets of cell membranes by simulations, which is then validated by the experiments. In combination with the energetic analysis through a newly developed theoretical model, the pore is found to present unstable, metastable, and stable states. Last, a quantitative correlation between simulations and theoretical analysis corroborates that the transition of transport patterns of the sandwiched GO can be fundamentally attributable to the membrane-pore states that contribute to various directional persistence.

As 2D NMs affect the phospholipid composition within cellular membranes, they further impact the characteristics and functionalities of membrane proteins. Interactions between specific nanomaterials and phospholipids influence the stability and structure of membrane proteins, altering their positioning and functional properties within the membrane. These alterations may affect the conformational transitions, ligand-binding capabilities, and efficiency of signal transduction for membrane proteins. Zhang et al. used graphene oxide passivated with PEG (GO-PEG) as a test bed, and presented a complete picture of the interaction between passivated 2D NMs and cell membranes to mediate integrin step-by-step activation by all-atom molecular dynamics simulation (Fig. 2F–H) [102]. This work provides insights into the potential de novo design and rational use of novel desirable nanomaterials at diverse bio–nano interfaces. A comprehensive understanding of these influences contributes to a deeper comprehension of the regulatory mechanisms by which two-dimensional nanomaterials modulate the functionality of membrane proteins, offering theoretical insights and technological support for designing targeted drug delivery systems and biosensors.

### 2D NMs-nucleic acids interactions

When considering the interaction between 2D NMs and nucleic acids (DNA and RNA), significant impacts on the properties and functionalities of these biomolecules arise. Studies indicate that interactions between two-dimensional nanomaterials, such as graphene, molybdenum disulfide, and nucleic acids, could induce alterations in the structure, stability, and functionality of DNA and RNA. Such interactions may lead to bending, twisting, or structural deformation of nucleic acid chains, potentially affecting the double-helix structure of DNA/RNA. For example, Zhang et al. designed and prepared a class of ultrathin two-dimensional MOF nanosheets, named Zr-BTB MOF nanosheets, composed of Zr − O clusters and 1,3,5-benzenetribenzoate by a bottom-up synthesis strategy [103]. The nano-bio interaction between DNA and Zr-BTB MOF nanosheets was deeply investigated by systematic molecular dynamics simulations and fluorescence spectroscopic analysis. The results of the dynamic adsorption process indicated that hydrogen bonding and π − π interactions played a key role in the difference in adsorption of ssDNA and dsDNA by the Zr-BTB MOF nanosheets. Nucleic acids form stabilizing π − π interactions differentially for different 2D NMs; this enables the design of complex nanomaterial heterostructures for nucleic acid handling and sequencing applications.

Additionally, the presence of 2D NMs in nano-bio interface might interfere with biological processes involving nucleic acids, such as unwinding, base pairing, transcription, and translation. In vivo, these influences could alter gene expression patterns or regulate fundamental cellular functions. Furthermore, the physical and chemical properties of nanomaterials, such as surface chemistry, size, and shape, also dictate the manner and extent of their interaction with nucleic acids. A deeper understanding of the interactions between two-dimensional nanomaterials and nucleic acids contributes to our comprehension of the potential applications of these materials in biomedicine and nanotechnology, including targeted gene delivery, diagnostics, and therapies.

Taken together, the nano-bio interface effects are determined by the unique structures and chemical properties of 2D NMs and the biological components, which include hydrophilic-hydrophobic interactions, π-π interactions, electrostatic interactions, surface reactivity and structural characteristics. Therefore, specific analysis of these phenomena requires a tailored approach using experimental methods and simulations to understand the intricate details. Experimental methods may face challenges related to reproducibility, microscopic mechanism, and ethical considerations, while simulations may encounter limitations in accurately representing the complex biological environments. Overcoming these challenges requires ongoing efforts to refine techniques, validate results, and bridge the gap between experimental observations and simulation predictions. For instance, the development of high-precision imaging tools like cryo-electron microscopy plays a pivotal role in observing and detecting nano-bio interactions. Utilizing such tools allows researchers to obtain detailed experimental results, which can then be used as input parameters for simulations to improve the reproducibility. This integrated approach enhances the accuracy and depth of our understanding of nano-bio interfacial effects, which can further guide the safe and effective application of 2D NMs in biological systems.

## Biomedical applications of 2D NMs in cancer treatment

Building upon the understanding of nano-bio interfacial effects, this section delves into the diverse applications of 2D NMs in cancer treatment, considering both their intrinsic characteristics and the aforementioned interface effects. This comprehensive approach examines their potential roles as candidate drugs, carriers for chemo/gene-therapeutic drugs, antigen carriers, immunomodulators, as well as agents for PTT and PDT. By intertwining the perspectives of nano-bio interactions and inherent nanomaterial traits, this exploration aims to provide a holistic understanding of the multifaceted contributions of 2D NMs in the realm of cancer treatment.

### 2D NMs as drug candidates

**Fig. 3:**
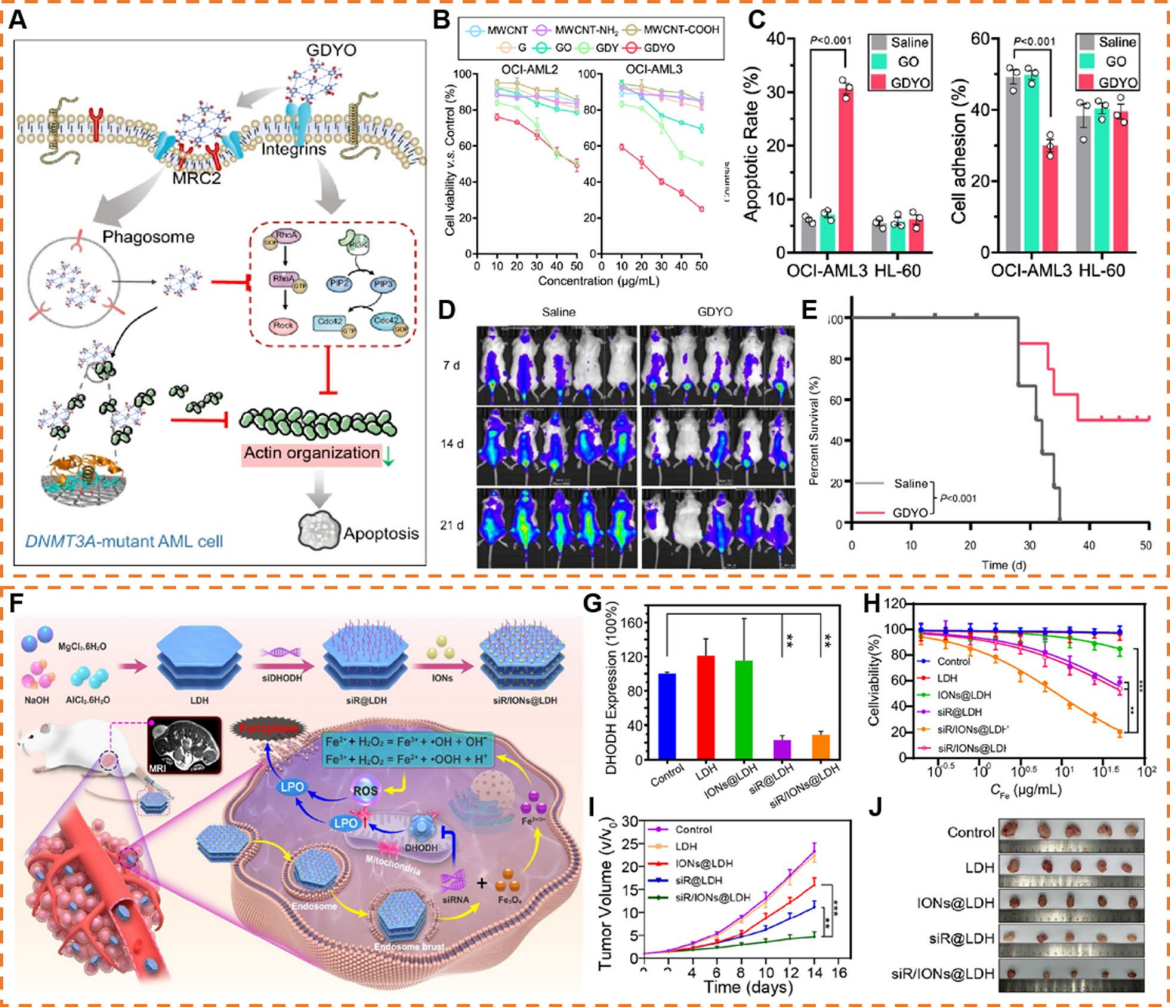
2D NMs as drug candidates and gene-therapeutic drugs carriers. **A** Illustration for GDYO therapy against DNMT3A-mutant AML cell. **B** Cell viability assay of DNMT3A-mutant AML cell (OCI-AML2 and OCI-AML3) treated with different carbon-based nanomaterials for 24 h. **C** Apoptotic rates (left) and cell adhesion assay on fibronectin (right) for DNMT3A-mutant AML cell (OCI-AML3) and DNMT3A-wildtype AML cell (HL-60) treated with GO/GDYO for 48 h. **D** IVIS spectrum images of mice with different treatments. **E** Kaplan–Meier survival curve of AML mice injected with saline or GDYO. (Reproduced with permission from Ref. [^106^], Copyright 2022, Springer Nature) **F** Schematic illustration of the construction and theranostic mechanism of the siR/IONs@LDH nanoplatform. **G** Protein expression levels of DHODH in 4T1 cells determined by a western blot assay. **H** Cell viability of 4T1 cells with different treatments. **I** Mean tumor volume of resected tumors when 4T1 tumor-bearing mice were sacrificed on day 14. **J** Photographs of resected tumors when 4T1 tumor-bearing mice were sacrificed on day 14. (Reproduced with permission from Ref. [^136^], Copyright 2023, American Chemical Society)

2D NMs’ direct cellular effect with specific cells or organelles may have unexpected outcomes, which leading to new opportunities for providing more efficient nanomedicine. For example, Ma group reported that 2D NMs could bind to cell surface receptors (e.g., integrins [104] and L-type calcium ion channel Ca_v_1.3 [105]) to activate specific signaling pathways and produced unique cellular responses which can be attributed to the cell membrane disturbance induced by 2D NMs. Recently, Wang et al. found that graphdiyne oxide (GDYO) could directly interact with integrin β2 (ITGB2) and c-type mannose receptor (MRC2) to endocytosis by DNA methyltransferase 3 A (DNMT3A)-mutant acute myeloid leukemia (AML) cells (Fig. 3A–C) [106]. Notably, GDYO could bind to actin and prevent actin polymerization, thus damaged actin cytoskeleton and led to AML cells apoptosis rather than other sensitive cells (e.g., neurons), which suggesting the promising anti-leukemia efficacy and suitable biosafety by GDYO (Fig. 3D, E). However, the pharmacokinetics of GDYO should also be explored for further clinical applications.

In addition, 2D NMs can also have direct effects on organelles and thus affect tumor cell progression. As an example, Shao et al. discovered that BP could directly destroy mitotic centrosomes and led to multipolar spindle formation, which was attributed to the impaired cohesion of outer pericentriolar material with centrosomes in early mitosis [107]. This study revealed that BP deactivated centrosomes kinase Polo-like Kinase 1 (PLK1) thus induced apoptosis due to mitotic arrest. It was worth noting that this effect produced by BP preferred to kill cancer cells rather than normal cells. On this basis, BP presented significant anti-tumor effect on a xenografted nude mouse model with Hela cells via measurement by tumor size, weight and tumor TdT-mediated dUTP nick end labelling (TUNEL) staining. This study revealed the unique biological mechanism and highlighted the great medical value of BP nanosheets.

### 2D NMs as chemo-therapeutic drugs carriers

In recent years, 2D NMs have garnered significant attention as drug delivery carriers in the field of cancer therapy. This is primarily attributed to their multiple advantages sourced from nano-bio interfacial effects, which include: (1) 2D NMs possess a large surface area due to the unique lamellar structure, facilitating efficient drug loading; (2) 2D NMs can be loaded with drugs through non-covalent or covalent interaction, the previous one refers to interact with hydrophobic drugs through π-π stacking or hydrophobic interactions, and the latter one refers that the chemical composition of some 2D NMs can exhibit specific reactions with certain drugs, thereby enhancing drug-loading capacity [108]. The loading of chemotherapeutic drugs onto 2D NMs has aroused widespread attention and has been extensively investigated. Among the tested anti-cancer drugs was doxorubicin (DOX), camptotehecin, platinum et al., via electrostatic adsorption or π-π stacking [109]. Table 1 shows examples of the interactions and loading capacity of different chemotherapeutic agents on various 2D NMs. Among these, DOX, a normally used chemotherapeutic agent in clinic, was always chosen as the model drug to test the potential of 2D NMs as drug delivery carriers.

**Table 1:** Representative examples of 2D NMs loaded with various chemo-therapeutic drugs

Drug	2D NMs	Nano- bio interfacial interactions	Loading capacity (%)	Refs.
Doxorubicin	NGO	π-π stacking; hydrophobic interactions	400	[114]
	rGO/dopa-MAL	π-π stacking	17	[115]
	PEG-BPEI-rGO	π-π stacking; hydrophobic interactions	100	[116]
	GO	π-π stacking; hydrophobic interaction	49	[117]
	GO	π-π stacking; hydrogen-bonding	33	[113]
	GO	π-π stacking; hydrophobic interaction	213	[118]
	P-LDH	Electrostatic interaction	734	[111]
	BP	Electrostatic interaction	950	[110]
	BP	hydrophobic interaction	187	[119]
	Antimonene-PEG	π-π stacking; hydrophobic interactions	150	[120]
	InSe-PEG	hydrophobic interactions	93.6	[121]
	POVK-MoS_2_	Acid-cleavable Schiff base linker	6.1	[62]
	MoS_2_-PEI-HA	Electrostatic interaction	33.6	[59]
	Ti_3_C_2_	Electrostatic interaction	84.2	[34]
	TaS_2_-PEG	Electrostatic interaction; π-π stacking; hydrophobic interactions	177	[122]
	Zirconium boride	borate esterfication	121	[123]
	Boron nanosheet	π-π stacking; hydrophobic interactions	114	[124]
	Silicene-BSA	π-π stacking; hydrophobic interactions	137	[125]
	Germanene-based nanosheet	hydrophobic interactions; electrostatic adsorption	112.5	[126]
	Lanthanum oxyiodide	π-π stacking	300	[127]
Camptotehecin-based	NGO-PEG	Van der Waals	10	[28]
	MoS_2_-PEG	π-π stacking; hydrophobic interactions	118	[58]
	NGO	π-π stacking; hydrophobic interactions	4.5	[114]
Docetaxel	GO	π-π stacking; hydrogen-bonding	37	[112]
	GO-PEG	π-π stacking; hydrophobic interactions	11.2	[128]
Platinum-based	BP	coordination interaction between Pt and P	77	[129]
5-fluorouracil	Silicon nanosheet	Electrostatic interaction	30.03	[130]
	LDH	Electrostatic interaction	22.6	[131]
AQ4N	Iron-based nanosheet	π-π stacking; electrostatic interaction	14.44	[132]
FX11	WS_2_	Van der Waals; hydrogen bonding interaction	21.9	[133]

2D NMs exhibits an extremely high specific surface area attribute to their lamellar structure. Chen et al. reported that BP, which had much higher surface to volume ratio due to the puckered lattice configuration, could encapsulate higher amounts of DOX (950% in weight) within the interlayer spaces via electrostatic interaction than the reported 2D NMs [110]. However, the drug loading capacity is largely restricted by multilayered 2D NMs’ interlayer spacing and the size of drug molecules. Based on this, Zhang et al. constructed an monolayered LDH nanosheet and maximized the loading capacity of DOX (734% in weight), which was almost seven times that of multilayered LDH nanosheet [111].

In addition to extremely high drug loading capacity, the chemical composition of 2D NMs can be modified with targeting moiety (e.g., folic acid, hyaluronic acid, RGD peptide) to achieve tumor targeted drugs delivery, while the inherent targeting functionalities of certain 2D NMs have been ignored [112–114]. For instance, Ma group discovered that GO with ultrasmall size (~ 40 nm) and a certain oxidization degree (26.1%) was hosted in the hydrophobic interior of membrane. Such a unique sandwiched structure made GO a potential carrier for membrane-specific drug delivery. Building upon this finding, we loaded vandetanib (VTB), which was the inhibitor of epidermal growth factor receptor, onto GO and known as GO-VTB. The maximum inhibitory concentration (IC50) of GO-VTB was ~ 1.25 μg/mL, which was nine- and six-fold lower than that of free VTB and classical liposome encapsulated VTB (lip-VTB), respectively [101]. This finding has significant implications for guiding the design of novel 2D NMs for precise drug delivery (e.g., targeting membrane receptors).

### 2D NMs as gene-therapeutic drugs carriers

The unique geometry of 2D NMs promotes efficient loading of genetic materials. Ji et al. reported the DNA loading efficiency of spherical nanoparticles and nanosheets made of the same material, silica. The DNA loading efficiency of silica nanosheets was 34%, which was ten times that of spherical silica nanoparticles, indicating that the shape of materials significantly influences their ability to carry genetic material [134].

Nucleic acids can be adsorbed in different positions of 2D NMs through hydrophobic, π-π stacking and electrostatic interactions. For an instance, Zeng et al. directly adsorbed permeability glycoprotein (P-gp) siRNA on the surface of BP in 66.7% ethanol and the adsorbed amount of small interfering RNA (siRNA) was almost 4.62 nmol/mg [70]. For multilayer 2D NMs, nucleic acid also could be loaded into the interlayer. For example, LDH exhibits superior anion exchange capacity, enabling intercalate any biomolecules with negative charges into its interlayer region [131]. More importantly, such a loading strategy offers remarkable protection against DNase I and protection against thermal damage [135]. On this basis, Chen et al. employed a LDH nanoplatform for co-delivering siRNA against dihydroorotate dehydrogenase (siR) with iron oxide nanoparticles (IONs) (Fig. 3F). The disability of dihydroorotate dehydrogenase-mediated ferroptosis defense could effectively sensitize tumor cells to toxic reactive oxygen species (ROS) via Fe^2+^-mediated Fenton reaction, synergically induced cancer cell death upon the accelerating of lipid peroxidation (LPO) accumulation (Fig. 3G–J) [136].

Nucleic acid loading capacity of 2D NMs can also be increased via chemical modification using cationic polymers (e.g., PEI, polypropylenimine (PPI)) to electrostatically attract negatively charged genetic materials [60, 71, 137–140]. In addition, cationic polymers endowed 2D NMs with improved transfection efficiency. This is attributed to their ability to recruit large amounts of protons to disrupt endosomal membrane, which is also known as ‘proton sponge effect’. However, some reports indicated that the cytotoxicity of PEI limited the gene loading efficiency of 2D NMs. In contrast to PEI, polyamidoamine (PAMAM) allowed a higher number of well exposed surface amine groups to link to siRNA and offered the advantage of biodegradability [141]. Yadav et al. modified PAMAM on the surface of GO-PEG through covalent reaction of carboxylic groups and primary amino groups using NHS/EDC to form GPD [141]. The negatively EPAC1 siRNA was bound to GPD through electrostatic, hydrophobic and π-π stacking interactions, neutralizing the positive charge of GPD and reducing cytotoxicity. GPD demonstrated superior transfection efficiency, significantly restraining cell migration, and exhibiting lower invasion compared to Lipofectamine 2000. These findings implied its potential role in preventing the progression and metastasis of breast cancer.

### 2D NMs as antigen carriers

The efficient loading of antigen is one of the crucial preconditions for cancer vaccine to effectively elicit immune responses. Similar to load drugs, there are different kinds of forces involved when antigens interact with 2D NMs. Hydrophobic, π-π stacking, electrostatic, van der Waals and hydrogen bonding are some of the important non-covalent interactions that enable the loading of antigen. Moreover, the crimpable sheet-like structure and flexible 2D backbone allowed for loading cargos through wrapping and folding [142]. Such a unique cargo loading of 2D NMs provides a new theoretical basis for the development of antigen carriers. In addition to non-covalent interactions, Li and co-workers demonstrated that covalent modifications could improve the loading efficiency of antigens. They modified ovalbumin (OVA) with several BP binding motifs including aromatic and amino groups, a screened tripeptide, phenylalanine-lysine-phenylalanine (FKF), significantly increased OVA loading after incubation. OVA showed a lower BP loading efficacy of 16.9%, while that of FKF-OVA was 69.8% [143].

**Fig. 4:**
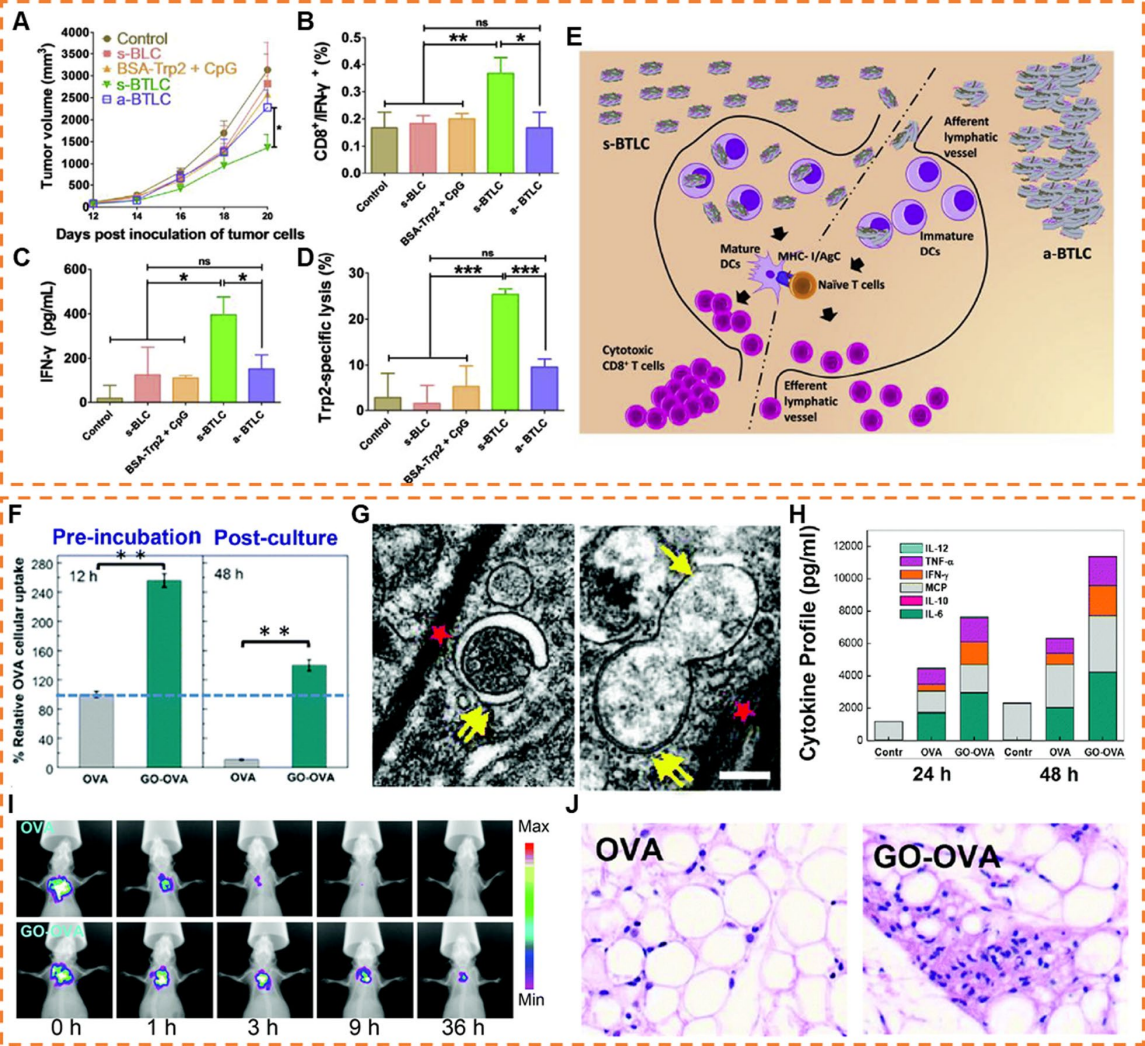
2D NMs as antigen carriers. **A** Tumor size changes post inoculation monitored every 2 days. **B** Proportion of CD3^+^/CD8^+^/IFN-γ.^+^ cells in splenocytes from vaccinated mice with the cells pulsed with Trp2 for 48 h. **C** The levels of IFN-γ in medium with splenocytes pulsed with Trp2 for 48 h. **D** In vivo CTL responses after vaccination with various NPs. **E** Schematic presentation for s-BTLC and a-BTLC to penetrate into dLNs. (Reproduced with permission from Ref. [^147^], Copyright 2018, Elsevier) **F** Flow cytometry (FACS) data showing the improved OVA internalization in BMDC with or without the assistance of GO after 12 h OVA pre-incubation and 48 h post-culture period. **G** Transmission electron microscopy images of autophagy procedure undergone by the GO pulsed DCs. Typical double-membrane autophagosomes (indicated by double-head arrows) were found in GO (indicated by red stars) treated BMDC. Scale bar: 200 nm. **H** Cytokine profile of the antigen-challenged BMDC at different coincubation (BMDC-CD8 T) times. **I** Comparison of the Cy5-OVA distribution with or without GO after subcutaneous administration in the hindneck of C57BL/6 mice. **J** Hematoxylin and eosin (H&E) staining of the APC recruitment in mice immunized with GO–OVA. (Reproduced with permission from Ref., [^148^] Copyright 2015, The Royal Society of Chemistry)

Given that immune responses predominantly occur within lymph nodes, the efficient delivery of antigen-loaded 2D NMs to these sites is pivotal. This involves primarily two modalities, with one approach being the direct transport of the antigen carriers along the lymphatic circulation into the lymph nodes (LNs) [144]. This approach is constrained by the size limitations of the carrier, with large particles (> 500 nm) remaining entrapped in extracellular matrix (ECM) rather than entering LNs [145]. However, some 2D NMs easily aggregated at the injection site after subcutaneous injection, which greatly limited the efficacy of many 2D NMs vaccines [146]. Given this, Zhang et al. controlled the aggregation status of LDH by coating BSA to prepare dispersed or aggregated BSA/LDH [147]. Compared to aggregated BSA/LDH, dispersed BSA/LDH induced almost 6.7 times more antigen-specific cytotoxic lymphocyte (CTL) activity in vivo (Fig. 4A–D). Such a difference was attributed to the easy penetration of dispersed BSA/LDH into draining LNs (dLNs) resulting in more dendritic cells (DCs) endocytosis and presented antigen epitopes to activate CD8^+^ T cells. In comparison, aggregated BSA/LDH rather remained in the injection site than penetrated into dLNs due to the larger size (Fig. 4E).

The other approach is to be captured by resident antigen-presenting cells (APCs) and then homing to LNs [144,145]. Hence, promoting the recruitments of APCs to the injection site is also of paramount importance. Ma group discovered a promising GO-based vaccine. Once this vaccine is taken up by DCs, the flat GO structure transforms, shifting into a folded shape. This folded morphology demonstrates its capability to serve as an antigen reservoir. Essentially, it can encase proteins like OVA within its folds, safeguarding them from degradation by enzymes (Fig. 4F and G) [148]. After subcutaneous injection of GO-OVA, the antigen signal remained 20% at 24 h in the injection site. Such durable antigen processing and presentation endowed APCs with more opportunities to elicit specific immune responses. In addition, GO-OVA induced DCs to secrete larger amount of interleukin-6 (IL-6) and monocyte chemotactic protein-1 (MCP-1) compared to free OVA, which could recruit more APCs (Fig. 4H–J). Compared to other 2D NMs-based vaccines, this platform was simpler and more practical without any extra addition of bio- or chemical stimulators. This work revealed the potential use of the unique bio- or physiochemical properties of 2D NMs in the development of high-performance cancer vaccines.

Considering the significant importance of cross-presentation in eliciting anti-tumor effects from vaccines, 2D NMs have also contributed to this aspect. For example, Xu et al. prepared a PEGylated reduced GO (RGO-PEG) sized 20 to 30 nm in diameter to load neoantigens and CpG [149]. Interestingly, RGO-PEG increased the intracellular ROS in APCs. This consequently resulted in an increased endolysosomal pH and greatly prevented the rapid degradation of antigens in endolysosomes, leading to the improved and prolonged antigen cross-presentation to CD8α^+^ T cells. Furthermore, autophagy was associated with antigen cross-presentation. Ma group has uncovered the folded-GO endocytosed by DCs induced autophagy of DCs through Beclin 1 and LC3 II mediated pathway. This induction results in the facile fusion of antigens released from GO with autophagosomes, thereby enhancing the cross-presentation to the CD8 T cells. This work revealed a crucial mechanism for autophagy based on 2D NMs.

### 2D NMs as immunomodulators

Given the macrophages comprise a significant portion of tumor mass, accounting for 30–50% of the cells, investigating macrophages within tumor microenvironment may hold the key to effective immunotherapy [150]. Upon the introduction of 2D NMs into the body, macrophages play a predominant role in processing these materials. Upon stimulation, macrophages release a myriad of cytokines and chemokines. These cytokines serve to activate and recruit other cells for the clearance of invading pathogens. Therefore, understanding the biological effects of the interaction between 2D NMs and macrophages is imperative.

Once 2D NMs are internalized by macrophages and enter the cytoplasm, they are inevitably prone to interacting with intracellular proteins and even organelles. For an instance, when GDYO nanosheet entered the cytoplasm of tumor-associated macrophages (TAMs), the signal transduction and activator of transcription (STAT3) protein were adsorbed onto the surface of GDYO to prevent STAT3 from entering the nucleus and polarize TAM [151]. To dissect the further mechanism, GDYO could not only specifically interact with STAT3 protein, but the unique physicochemical properties of activating pro-inflammatory pathways (mitogen-activated protein kinase (MAPK), interleukin-1 (IL-1) processing and Toll-like receptor (TLR) signaling pathways) [152]. Importantly, the anti-tumor effects in mice were consistent with the involvement of both macrophage and CD8^+^ T cells, demonstrating that macrophages could also be a key therapeutic target for 2D NMs antitumor immunotherapy.

**Fig. 5:**
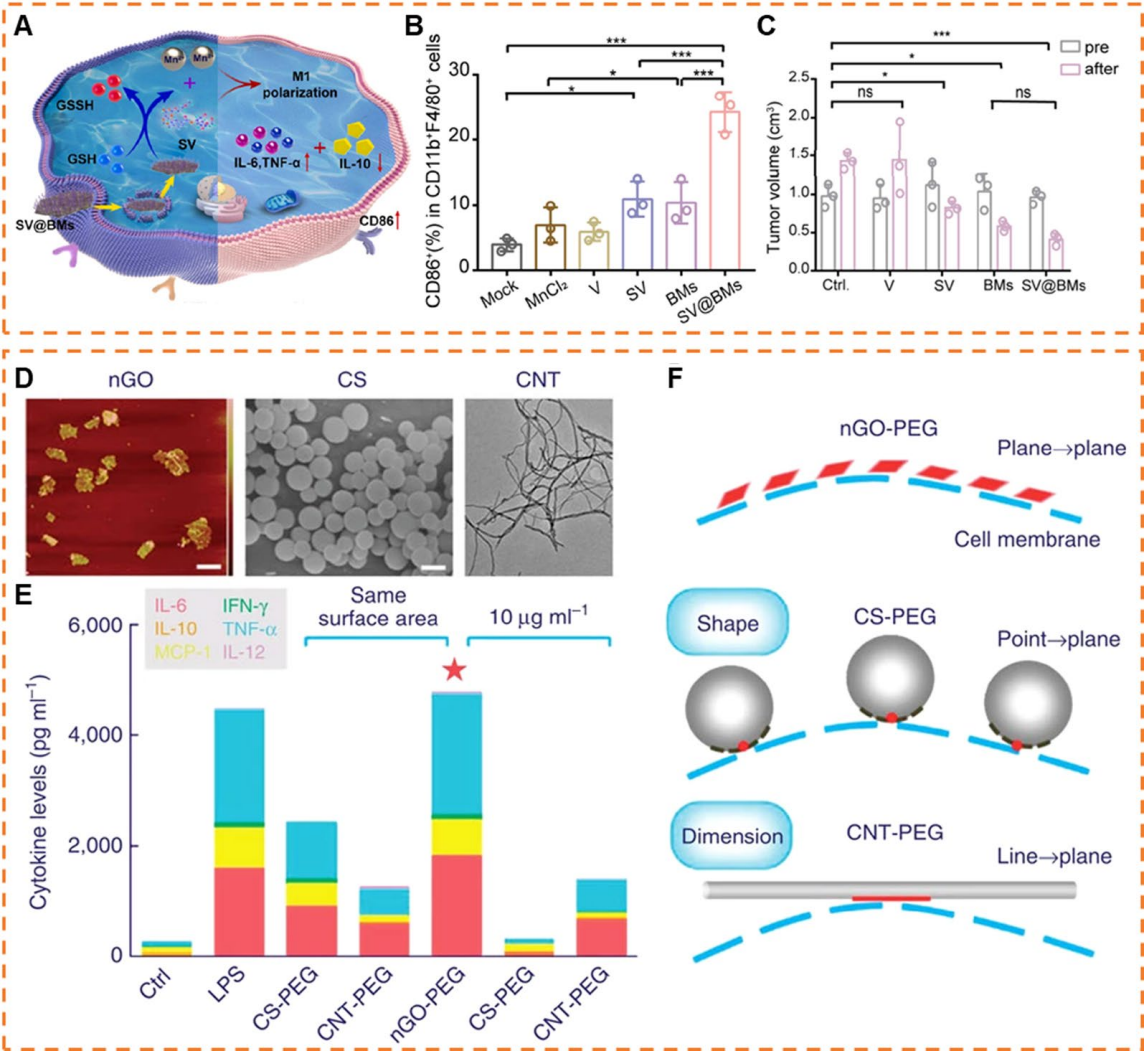
2D NMs as immunomodulators. **A** Schematic illustration of macrophage polarization by SV@BMs. **B** Percentages of M1-like macrophages in each group after 24 h. **C** The volume of the tumor measured by T_2_-MRI. “Pre” means the initial tumor size before treatment and “after” means the tumor size at the end time point of observation. (Reproduced with permission from Ref. [^153^], Copyright 2022, Elsevier) **D** Atomic force microscopic (AFM), scanning electron microscopic (SEM) and TEM images of graphene oxide, carbon spheres and carbon nanotubes, respectively. Scale bars: 200 nm. **E** Inflammatory cytokines secreted by macrophages when exposed to different PEGylated carbon nanomaterials, normalized by surface area and concentration. **F** Conjecture for interaction modes between carbon nanomaterials and cell membranes resulting in different levels of cytokine secretion. (Reproduced with permission from Ref. [^104^], Copyright 2017, Springer Nature)

Considering that 2D NMs are readily engulfed and degraded by macrophages, it is worthwhile to investigate the impact of the degradation products on macrophages. On this basis, Liang et al. constructed MnO_2_ nanosheets-based platform [153]. This platform could be decomposed by GSH to release Mn^2+^ and produce reactive oxygen species (ROS) from H_2_O_2_ via Mn^2+^-mediated Fenton-like reactions (Fig. 5A). The ROS production polarized macrophage and produced pro-inflammatory cytokines depending on the activation of NF-κB signal pathway, ultimately inducing apoptosis of osteosarcoma cells (Fig. 5B, C). After combining with Toll-like receptors 7/8 (TLR7/8) agonist, this approach significantly inhibited lung metastasis due to activation of systemic immunity via intra-tumoral injection. However, the systemic administration is more common than local injection in terms of clinical applications. Therefore, further exploration of the effects of systemic administration of this platform should be conducted.

PEGylated 2D NMs have been demonstrated to impede the internalization of macrophages. In principle, this hindrance can prevent macrophage activation and subsequent immune responses. In contrast, Ma group uncovered macrophages exhibited a specific nano-bio interfacial effect in response to PEGylated GO (nGO-PEG) than initially hypothesized [104]. While nGO-PEG evaded internalization by macrophages, it still elicited a heightened release of cytokines to activate macrophages by physically interacting with cell membranes (Fig. 5D–F). This behavior enhanced the interactions between nGO-PEG and integrin α_v_β_8_, subsequently activating of focal adhesion kinase (FAK)-related intracellular signaling pathways that ultimately led to macrophage activation. However, whether the activation induced by 2D NMs leads to further inflammatory or immunological responses will require additional in vivo experiments to verify.

### 2D NMs as PTT agents

In recent times, 2D NMs have emerged as highly promising photothermal agents owing to their ultrathin structure, exceptional photothermal conversion efficiency (PCE), and distinctive optoelectronic properties. Under NIR light irradiation, these 2D NMs manifest plasmonic effects that facilitate the efficient conversion of light energy into thermal energy. As a result, they have found widespread application as PTT agents, as illustrated in Table 2.

**Table 2:** Reported extinction coefficient and PCE of various 2D NMs

2D NMs	NIR (nm)	Extinction coefficient (L g^−1^ cm^−1^)	PCE (%)	Refs.
Silicene	808	15.6	22.50	[154]
	1064	16.0	36.09	[155]
AsP	1064		37.6	[156]
Boron nanosheet	808		42.5	[124]
Palladium	808		49.80	[157]
Zirconium carbide	808	8.8	52.1	[158]
	1064	8.3	62.1	[158]
BP	808	20.6	38.8	[159]
Ti_3_C_2_Tx	808	1.70	24.96	[160]
Ti_3_C_2_	1064	4.81	31.78	[161]
Ti_3_C_2_	808	25.2	30.6	[44]
Ti_3_C_2_	808	28.6	58.3	[34]
Ti_3_CN	808	36.6	36.5	[162]
	1064	43.5	32.1	[162]
Nb_2_C	1064	21.11	27.03	[163]
Nb_2_C	808	37.6	36.5	[42]
	1064	35.4	46.65	[42]
Mo_2_C	808	18.0	24.5	[36]
	1064	12.3	43.3	[36]
MnPc	730	49.7	72.3	[164]
MnO_2_	808	5.0	21.4	[165]
MnO_2_	808	2.68	21.00	[91]
MnOx/TiO_2_-GR	808	10.1	18.4	[166]
TeSe	808		99.1	[167]
Heterostructure bismuth selenide and tungsten selenide	808		40.75	[168]

**Fig. 6:**
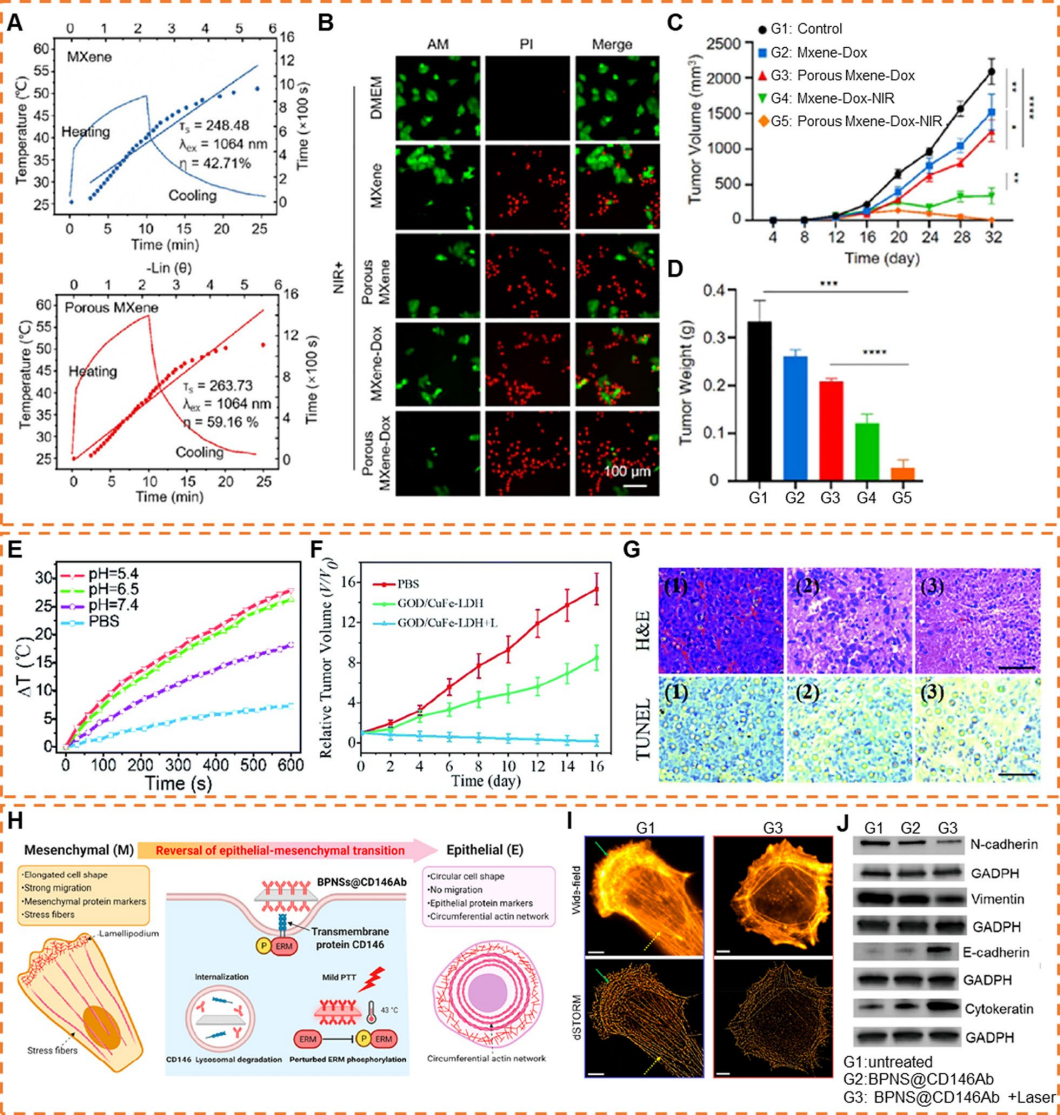
2D NMs as PTT agents. **A** Photothermal conversion efficiency of MXene (η = 42.71%) and porous MXene (η = 59.16%). **B** AM/PI staining of live, dead cells and apoptotic cells ratio after NIR irradiation. **C** Time-dependent tumor growth curves after different treatments. **D** Tumor weights of different groups after the treatments. (Reproduced with permission from Ref. [^169^], Copyright 2023, American Chemical Society) **E** Photothermal heating curves of PBS and GOD/CuFe-LDHs at pH 7.4, 6.5 and 5.4. **F** Hela tumor growth curves with various drug treatments. **G** H&E and TUNEL stained tumor tissue slices from different groups of mice after 16 d post-treatment. Scale bar: 100 μm. (Reproduced with permission from Ref. [^170^], Copyright 2021, The Royal Society of Chemistry) **H** Schematic illustration of reversing the epithelial − mesenchymal transition (EMT) in metastatic, mesenchymal-type cancer cells via targeting a transmembrane EMT inducer, CD146, using engineered black phosphorus nanosheets (BPNSs) and a mild photothermal treatment. **I** Wide-field and dSTORM images of ventral actin organization in untreated and treated cells. Scale bars: 5 μm. **J** Western blot analyses of EMT-related molecular markers. GAPDH was used as the housekeeping protein. (Reproduced with permission from Ref. [^171^], Copyright 2023, American Chemical Society)

Most photothermal agents requires high irradiation intensities which may cause harm to normal tissues; hence, it is critical to improve the PCE while reducing the irradiation intensity. The PCE of 2D NMs is limited by different factors, such as the structure of 2D NMs, their absorption of NIR light can be enhanced by changing their structure. As a paradigm, Zhu et al. discovered that incorporation of more electronegative N enhanced the electron counts of MXenes, leading to an improvement of localized surface plasmon resonance (LSPR) [162]. Such an enhancement allowed Ti_3_CN an extinction coefficient of up to 43.5 L g^−1^ cm^−1^ at 1064 nm, which was superior to Ti_3_C_2_ of 17.0 L g^−1^ cm^−1^. Furthermore, the porous structure can also enhance the surface plasmon resonance (SPR) effect thereby achieving improved optical absorption performance [169]. Hui et al. constructed porous MXene and validated its higher PCE of 59.16%, surpassing that of pristine MXene of 42.71% (Fig. 6A–D) [169]. Through the finite difference time domain method simulation and chemical composition analysis, it could be observed that the porous structure induced SPR effect. Additionally, the dangling bonds and defects formed by C–Co, Nb–O, etc., could also serve as exciton-phonon recombination centers to enhance PCE. This platform offers novel therapeutic options for osteosarcoma.

In addition to optimizing the structure during the preparation process to enhance PCE, in vivo modifications of 2D NMs can be carried out based on the characteristics of the tumor microenvironment, selectively augmenting their PCE within the tumor. Take an illustration, LDHs can undergo reorganization leading to the generation of numerous defects under acidic conditions in tumor. This process induces the formation of efficient acid-enhanced NIR photothermal conversion through the generation of photogenerated electron–hole pairs. Hu et al. constructed a Cu-based LDH platform with the PCE of 46.0% at pH 7.4, while significantly increased to 75.1% and 83.2% at pH 6.5 and 5.4, respectively, surpassing the performance of the majority of state-of-the-art nanoparticle-based systems (Fig. 6E–G) [170]. Such a novel pH-response LDH-based platform holds great promise in PTT.

Although PTT has demonstrated promising therapeutic outcomes via optimizing PCE of 2D NMs, its clinical implementation still encounters certain challenges. For instance, a considerable number of disseminated cells persist at the tumor periphery after PTT, contributing to tumor metastasis. In light of this issue, some researchers have proposed the use of mild photothermal therapy to modulate the phenotype of cancer cells, such as reversing epithelial-mesenchymal transition (EMT) and attenuate cancer stemness [171, 172]. As an illustration, Liu et al. prepared CD146 antibody-functionalized BP nanosheets, which facilitated the internalization of nanosheets by CD146 overexpressed tumor cells [171]. Subsequently, this process downregulated the membrane CD146 via directing transmembrane CD146 to a lysosomal degradation pathway. Furthermore, upon near-infrared laser irradiation, the mild temperature (~ 43℃) interrupted ERM phosphorylation, thereby reducing the “bridges” that link RhoGDlα to the remaining transmembrane CD146 (Fig. 6H). These events led to the downregulation of RhoA activation and its downstream effectors, such as cell migration and EMT protein transcription, thus reversed the mesenchymal-type cancer cells to an epithelial phenotype (Fig. 6I, J). Pretreating tumors with this approach could impede the metastasis of cancer cells, addressing the limitations of traditional surgical resection and PTT.

### 2D NMs as PDT agents

PDT stands as a clinically endorsed antitumor treatment characterized by precise controllability, minimal invasiveness, and absence of drug resistance. This therapeutic approach employs light in conjunction with photosensitizers (PSs) to initiate the generation of deleterious ROS, ultimately leading to the demise of tumor cells [173]. Certain characteristics of 2D NMs confer upon them superior PSs properties compared to other nanomaterials. For example, PEG modified 2D MOF nanosheet composed of Zn^2+^ and tetrakis(4-carboxyphenyl) porphyrin (TCPP) demonstrated greatly enhanced ROS production compared to their particulate counterpart [174]. This phenomenon was attributed to the higher specific surface area, which allowed much easier accessibility to surrounding oxygen molecules and more efficient interactions.

**Fig. 7:**
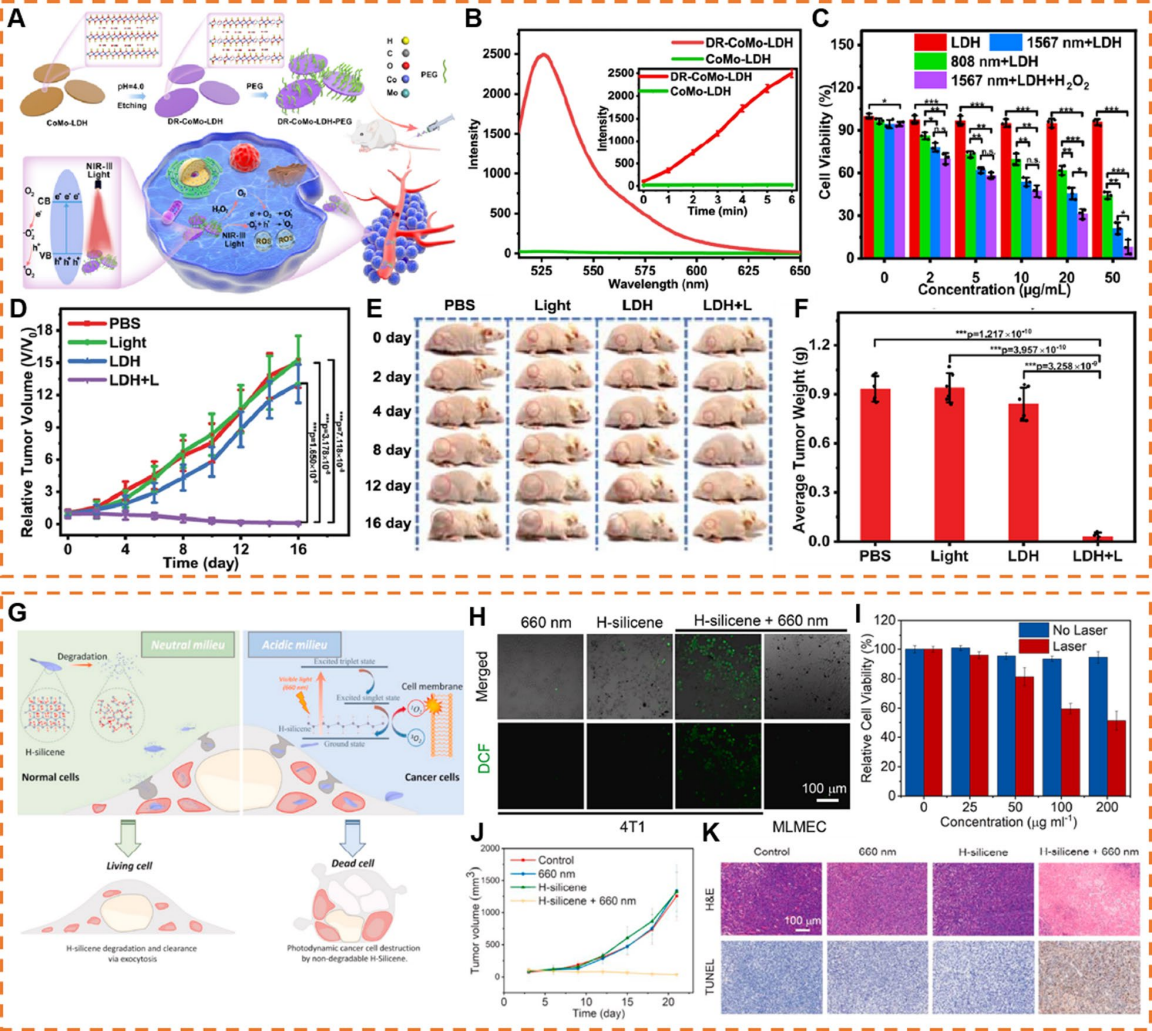
2D NMs as PDT agents. **A** Schematic illustration of the defect engineering of CoMo-LDH nanosheets, surface modification with PEG and its application in NIR-III PDT. **B** The fluorescence intensity of SOSG in presence of the CoMo-LDH and DR-CoMo-LDH nanosheets in H_2_O under 1567 nm laser irradiation (0.5 W cm.^−2^). **C** In vitro cytotoxicity profiles of 4T1 cells incubated with DR-CoMo-LDH-PEG under different conditions. **D** Tumor growth curves of mice after various treatments. **E** Representative photographs of mice with treatments at various time points. **F** corresponding average weight of tumors taken on Day 16. (Reproduced with permission from Ref. [^177^], Copyright 2022, Springer Nature) **G** Schematic illustration of semiconducting 2D H-silicene nanosheets of selective biodegradability for in the photodynamic tumor-specific therapy. **H** Confocal fluorescence images of 4T1 cells treated with DCFH-DA after different treatments. **I** Relative viabilities of 4T1 cells after incubated with varied concentrations of H-silicene nanosheets with or without 660 nm irradiation. **J** Tumor growth curves of 4T1 tumor model received different treatments. **K** H&E and TUNEL for 4T1 tumor tissues in mice models after different treatments in day 21. (Reproduced with permission from Ref. [^179^], Copyright 2021, Elsevier)

Several parameters such as size, morphology, defects of 2D NMs are intimately associated with the treatment outcome and efficacy of PDT. For example, reducing the layer thickness of 2D NMs substantially enhances the ROS generation. This is attributed to the simultaneous widening of the bandgap and the reduction of diffusion distance, which results in more efficient transport of electrons and holes to the material surface [175, 176]. Moreover, defects have been proven to affect the PDT performance of 2D NMs. Shen et al. constructed defect-rich CoMo-LDH (denoted as DR-CoMo-LDH) via simple acid treatment (Fig. 7A–F) [177]. After two hours etching, DR-CoMo-LDH exhibited excellent ROS production performance, which is about 97 times of pristine Co-Mo-LDH. Upon analysis of its structure, it was evident that DR-CoMo-LDH possessed much richer oxygen vacancies (OVs), which significantly enhanced the possibility of photogenerated electron–hole pairs to react with O_2_ to generate ROS.

Although the majority of 2D NMs tend to accumulate in the tumor region following local or systemic administration, a fraction of those persists within normal tissues. To mitigate off-target effects, most strategies involve the modification of targeting moieties (e.g., folic acid, hyaluronic acid, RGD peptide). Additionally, there is a growing focus on the responsive design of 2D NMs based on the tumor microenvironment, such as the GSH or the low pH, confining their functionality exclusively to tumor cells. As an illustration, Zhang et al. prepared copper-bacteriochlorin nanosheet (denoted as Cu-TBB) which could be hydrolyzed by GSH in tumor microenvironment to release Cu^2+^ and TBB [178]. The released TBB can generate ROS upon 750 nm laser irradiation. Along with Cu-triggered conversion of endogenous O_2_ to O_2_^**−**^**·**, this nanosheet could activate gasdermin D-mediated pyroptosis and promote the release of inflammatory cytokines, thus enhancing DC maturation and T lymphocyte priming, ultimately activating systemic adaptive immune response to inhibit the progression and metastasis of distant tumors.

Diverging from traditional therapeutic strategies that involve the development of pH-responsive biodegradable agents within physical tumors, Xu et al. developed H-silicene nanosheet featured acid pH-augmented structural stability but neutral pH-induced degradability, which might be due to the breakage of SiHn and SiH bonds in H-silicene (Fig. 7G) [179]. Based on the density functional theory, under 660 nm laser irradiation, electrons in H-silicene nanosheets were excited from the ground state to the excited triplet state, subsequently transitioning to the excited singlet state. This process was followed by the generation of singlet oxygen (^1^O_2_) from the ground-state triplet oxygen (^3^O_2_) on the H-silicene nanosheet, concomitant with the transfer of electrons from the excited singlet state to the ground state. Benefiting the high biocompatibility and ROS generation capacity, the potential in vitro and in vivo anti-tumor efficiency of H-silicene against 4T1 cancer, which provided an opportunity for cancer treatment while minimized side effects of H-silicene nanosheets in normal tissues (Fig. 7H–K).

## Conclusion and perspective

The nano-bio interface effects of 2D NMs hold significant promise in advancing anti-cancer therapies within the biomedical field. These materials have demonstrated remarkable potential in oncology due to their unique interactions with biological systems. The precise manipulation of the nano-bio interface allows for tailored drug delivery, enhanced imaging, and targeted therapy. 2D NMs exhibit notable advantages such as high surface area, tunable physicochemical properties, and specific binding capabilities to biomolecules, enabling efficient drug loading and controlled release. Additionally, their ability to induce photothermal or photodynamic effects can be utilized for localized tumor destruction and improved therapeutic outcomes. With ongoing research, these NMs show potential for synergistic multimodal therapies, combining multiple treatment modalities to overcome drug resistance and improve overall treatment efficacy.

However, to fully capitalize on the potential of these interfaces in biomedical applications, several challenges must be addressed, such as the biocompatibility and long-term safety of 2D NMs. The biocompatibility of 2D NMs is highly dependent on their physicochemical properties, including size, charge, surface properties and chemical composition, thereby regulating the nano-bio interface effects between 2D NMs and cells [180–184]. Without precise control, undesired outcomes such as inflammation, hypersensitivity reactions, or even life-threatening situations may arise [181, 185, 186]. PEGylation, a prevalent modification method, significantly enhances the biocompatibility of 2D NMs and confers them with an invisibility effect, prolonging their circulation time in the bloodstream. However, studies indicate that PEGylated nanoparticles may induce anti-PEG immunoglobulin M, potentially accelerating blood clearance after multiple administrations [187]. Moreover, long-chain hydrophilic polymers like PEG, while improving biocompatibility, can cause steric hindrance, reducing the activity of protein drugs. One solution is that considering the use of biodegradable polymers as modification agents. These materials could provide a balance between biocompatibility and avoiding long-term steric hindrance issues associated with PEGylation. Furthermore, advanced drug delivery systems can be integrated with passive NM modifications, endowing the capacity of targeted delivery mechanisms, triggered release, or combination therapies. In addition, immunomodulatory agents can be incorporated within the NM structure to actively regulate the immune response and prevent adverse reactions, such as anti-PEG immunoglobulin M production.

Although current research has investigated the in vivo and in vitro biocompatibility of 2D NMs and demonstrated short-term biocompatibility, a systematic evaluation of long-term safety of these materials is crucial for further expanding their practical applications in the field of biomedicine. On this basis, it is essential to evaluate the pharmacokinetics of 2D NMs to assess their metabolism and excretion [188]. Traditional pharmacokinetics relies on methods like inductively coupled plasma and Raman spectroscopy to analyze the concentration of 2D NMs over time in blood, organs, urine, and feces [189]. Nowadays, the in vivo tracking methods have been employed for real-time, non-invasive, and highly sensitive analysis of pharmacokinetics. These techniques encompass X-ray absorption near-edge spectroscopy, positron emission tomography et al. [190,191]. The research on pharmacokinetics contributes to the development of safer administration strategies, which is indispensable for the clinical translation of 2D NMs.

To realize the full potential of these advancements, it is imperative to further nurture interdisciplinary collaborations and invest in extensive research endeavors. Addressing fundamental challenges such as biocompatibility, long-term safety, and optimizing the interactions between nanomaterials and biological systems requires concerted efforts, such as the effective integration of simulation and experimental methods, improving the spatiotemporal resolution of experiments, and advancing multiscale simulation algorithms et al. Robust scientific investigations into the intricate dynamics at these interfaces, elucidating the underlying mechanisms governing cellular responses, and ensuring stringent safety standards are paramount to accelerate the translation of these innovations from the laboratory to clinical applications.

## Data Availability

The data used to support this review are included within the article.

## References

[1] Yu S, Zhang C, Yang H (2023). Two-dimensional metal nanostructures: from theoretical understanding to experiment. Chem Rev.

[2] Zhang X, Gong C, Akakuru O, Su Z, Wu A, Wei G (2019). The design and biomedical applications of self-assembled two-dimensional organic biomaterials. Chem Soc Rev.

[3] Kopp M, Kollenda S, Epple M (2017). Nanoparticle-protein interactions: therapeutic approaches and supramolecular chemistry. Acc Chem Res.

[4] Zhang H (2015). Ultrathin two-dimensional nanomaterials. ACS Nano.

[5] Singh AP, Biswas A, Shukla A, Maiti P (2019). Targeted therapy in chronic diseases using nanomaterial-based drug delivery vehicles. Signal Transduct Target Ther.

[6] Vankayala R, Hwang KC (2018). Near-infrared-light-activatable nanomaterial-mediated phototheranostic nanomedicines: an emerging paradigm for cancer treatment. Adv Mater.

[7] Weissleder R, Nahrendorf M, Pittet MJ (2014). Imaging macrophages with nanoparticles. Nat Mater.

[8] Park SM, Aalipour A, Vermesh O, Yu JH, Gambhir SS (2017). Towards clinically translatable in vivo nanodiagnostics. Nat Rev Mater.

[9] Gu Z, Zhu S, Yan L, Zhao F, Zhao Y (2019). Graphene-based smart platforms for combined cancer therapy. Adv Mater.

[10] Lin H, Chen Y, Shi J (2018). Insights into 2D MXenes for versatile biomedical applications: current advances and challenges ahead. Adv Sci (Weinh).

[11] Yadav V, Roy S, Singh P, Khan Z, Jaiswal A (2019). 2D MoS2 -based nanomaterials for therapeutic, bioimaging, and biosensing applications. Small.

[12] Zhu S, Gong L, Xie J, Gu Z, Zhao Y (2017). Design, synthesis, and surface modification of materials based on transition-metal dichalcogenides for biomedical applications. Small Methods.

[13] Anju S, Ashtami J, Mohanan PV (2019). Black phosphorus, a prospective graphene substitute for biomedical applications. Mater Sci Eng C Mater Biol Appl.

[14] Qiu M, Singh A, Wang D, Qu J, Swihart M, Zhang H, Prasad PN (2019). Biocompatible and biodegradable inorganic nanostructures for nanomedicine: silicon and black phosphorus. Nano Today.

[15] Sun Y, Gao S, Lei F, Xiao C, Xie Y (2015). Ultrathin two-dimensional inorganic materials: new opportunities for solid state nanochemistry. Acc Chem Res.

[16] Chen SH, Bell DR, Luan B (2022). Understanding interactions between biomolecules and two-dimensional nanomaterials using in silico microscopes. Adv Drug Deliv Rev.

[17] Roy S, Aastha, Deo KA, Dey K, Gaharwar AK, Jaiswal A (2023). Nanobio interface between proteins and 2D nanomaterials. ACS Appl Mater Interfaces.

[18] Roy S, Deo KA, Singh KA, Lee HP, Jaiswal A, Gaharwar AK (2022). Nano-bio interactions of 2D molybdenum disulfide. Adv Drug Deliv Rev.

[19] Tu Z, Guday G, Adeli M, Haag R (2018). Multivalent interactions between 2D nanomaterials and biointerfaces. Adv Mater.

[20] Geim AK (2009). Graphene: status and prospects. Science.

[21] Chae HK, Siberio-Pérez DY, Kim J, Go Y, Eddaoudi M, Matzger AJ, O'Keeffe M, Yaghi OM (2004). A route to high surface area, porosity and inclusion of large molecules in crystals. Nature.

[22] Ma R, Zhou Y, Bi H, Yang M, Wang J, Liu Q, Huang F (2020). Multidimensional graphene structures and beyond: unique properties, syntheses and applications. Prog Mater Sci.

[23] Dreyer D, Park S, Bielawski C, Ruoff R (2009). The chemistry of graphene oxide. Chem Soc Rev.

[24] Zhao X, Tao G, Gong X, Yang X, Ge H, Wang J (2019). Dual engineering interface-driven complementary graphene oxide-protein dimer supramolecular architecture enables nucleus imaging and therapy. ACS Appl Bio Mater.

[25] Diez-Pascual AM, Diez-Vicente AL (2016). Poly(propylene fumarate)/polyethylene glycol-modified graphene oxide nanocomposites for tissue engineering. ACS Appl Mater Interfaces.

[26] Altinbasak I, Jijie R, Barras A, Golba B, Sanyal R, Bouckaert J, Drider D, Bilyy R, Dumych T, Paryzhak S (2018). Reduced graphene-oxide-embedded polymeric nanofiber mats: an “on-demand” photothermally triggered antibiotic release platform. ACS Appl Mater Interfaces.

[27] Luo Z, Yang D, Qi G, Yuwen L, Zhang Y, Weng L, Wang L, Huang W (2015). Preparation of highly dispersed reduced graphene oxide decorated with chitosan oligosaccharide as electrode material for enhancing the direct electron transfer of escherichia coli. ACS Appl Mater Interfaces.

[28] Liu Z, Robinson JT, Sun X, Dai H (2008). PEGylated nanographene oxide for delivery of water-insoluble cancer drugs. J Am Chem Soc.

[29] Lin J, Chen X, Huang P (2016). Graphene-based nanomaterials for bioimaging. Adv Drug Deliv Rev.

[30] Naguib M, Kurtoglu M, Presser V, Lu J, Niu J, Heon M, Hultman L, Gogotsi Y, Barsoum MW (2011). Two-dimensional nanocrystals produced by exfoliation of Ti3 AlC2. Adv Mater.

[31] Anasori B, Lukatskaya MR, Gogotsi Y (2017). 2D metal carbides and nitrides (MXenes) for energy storage. Nat Rev Mater.

[32] Barsoum MW (2000). The MN+1AXN phases: a new class of solids: thermodynamically stable nanolaminates. Prog Solid State Chem.

[33] Anasori B, Xie Y, Beidaghi M, Lu J, Hosler BC, Hultman L, Kent PRC, Gogotsi Y, Barsoum MW (2015). Two-dimensional, ordered, double transition metals carbides (MXenes). ACS Nano.

[34] Liu G, Zou J, Tang Q, Yang X, Zhang Y, Zhang Q, Huang W, Chen P, Shao J, Dong X (2017). Surface modified Ti3C2 MXene nanosheets for tumor targeting photothermal/photodynamic/chemo synergistic therapy. ACS Appl Mater Interfaces.

[35] Yin H, Guan X, Lin H, Pu Y, Fang Y, Yue W, Zhou B, Wang Q, Chen Y, Xu H (2020). Nanomedicine-enabled photonic thermogaseous cancer therapy. Adv Sci (Weinh).

[36] Feng W, Wang R, Zhou Y, Ding L, Gao X, Zhou B, Hu P, Chen Y (2019). Ultrathin molybdenum carbide MXene with fast biodegradability for highly efficient theory-oriented photonic tumor hyperthermia. Adv Func Mater.

[37] Lin H, Wang Y, Gao S, Chen Y, Shi J (2018). Theranostic 2D tantalum carbide (MXene). Adv Mater.

[38] Dai C, Lin H, Xu G, Liu Z, Wu R, Chen Y (2017). Biocompatible 2D titanium carbide (MXenes) composite nanosheets for pH-responsive MRI-guided tumor hyperthermia. Chem Mater.

[39] Tao W, Kong N, Ji X, Zhang Y, Sharma A, Ouyang J, Qi B, Wang J, Xie N, Kang C (2019). Emerging two-dimensional monoelemental materials (Xenes) for biomedical applications. Chem Soc Rev.

[40] Xing C, Chen S, Liang X, Liu Q, Qu M, Zou Q, Li J, Tan H, Liu L, Fan D, Zhang H (2018). Two-dimensional MXene (Ti3C2)-integrated cellulose hydrogels: toward smart three-dimensional network nanoplatforms exhibiting light-induced swelling and bimodal photothermal/chemotherapy anticancer activity. ACS Appl Mater Interfaces.

[41] Tang W, Dong Z, Zhang R, Yi X, Yang K, Jin M, Yuan C, Xiao Z, Liu Z, Cheng L (2019). Multifunctional two-dimensional core-shell MXene@Gold nanocomposites for enhanced photo-radio combined therapy in the second biological window. ACS Nano.

[42] Lin H, Gao S, Dai C, Chen Y, Shi J (2017). A two-dimensional biodegradable niobium carbide (MXene) for photothermal tumor eradication in NIR-I and NIR-II biowindows. J Am Chem Soc.

[43] Ren X, Huo M, Wang M, Lin H, Zhang X, Yin J, Chen Y, Chen H (2019). Highly catalytic niobium carbide (MXene) promotes hematopoietic recovery after radiation by free radical scavenging. ACS Nano.

[44] Lin H, Wang X, Yu L, Chen Y, Shi J (2017). Two-dimensional ultrathin mxene ceramic nanosheets for photothermal conversion. Nano Lett.

[45] Rasmussen FA, Thygesen KS (2015). Computational 2D materials database: electronic structure of transition-metal dichalcogenides and oxides. J Phys Chem C.

[46] Wang QH, Kalantar-Zadeh K, Kis A, Coleman JN, Strano MS (2012). Electronics and optoelectronics of two-dimensional transition metal dichalcogenides. Nat Nanotechnol.

[47] Zhao W, Ribeiro RM, Eda G (2015). Electronic structure and optical signatures of semiconducting transition metal dichalcogenide nanosheets. Acc Chem Res.

[48] Li H, Lu G, Wang Y, Yin Z, Cong C, He Q, Wang L, Ding F, Yu T, Zhang H (2013). Mechanical exfoliation and characterization of single- and few-layer nanosheets of WSe2, TaS2, and TaSe2. Small.

[49] Nguyen EP, Daeneke T, Zhuiykov S, Kalantar-zadeh K (2016). Liquid exfoliation of layered transition metal dichalcogenides for biological applications. Curr Protoc Chem Biol.

[50] Zhao W, Tan X, Jiang J, Liu F, Mu T (2017). Highly efficient, green, and scalable β-cyclodextrin-assisted aqueous exfoliation of transition-metal dichalcogenides: MoS2 and ReS2 nanoflakes. Chem Asian J.

[51] Chou SS, Kaehr B, Kim J, Foley BM, De M, Hopkins PE, Huang J, Brinker CJ, Dravid VP (2013). Chemically exfoliated MoS2 as near-infrared photothermal agents. Angew Chem Int Ed Engl.

[52] Lee YH, Zhang XQ, Zhang W, Chang MT, Lin CT, Chang KD, Yu YC, Wang JT, Chang CS, Li LJ, Lin TW (2012). Synthesis of large-area MoS2 atomic layers with chemical vapor deposition. Adv Mater.

[53] Zhang Y, Yao Y, Sendeku MG, Yin L, Zhan X, Wang F, Wang Z, He J (2019). Recent progress in CVD growth of 2D transition metal dichalcogenides and related heterostructures. Adv Mater.

[54] Feng Z, Liu X, Tan L, Cui Z, Yang X, Li Z, Zheng Y, Yeung KWK, Wu S (2018). Electrophoretic deposited stable Chitosan@MoS2 coating with rapid in situ bacteria-killing ability under dual-light irradiation. Small.

[55] Yang G, Zhang R, Liang C, Zhao H, Yi X, Shen S, Yang K, Cheng L, Liu Z (2018). Manganese dioxide coated WS2 @Fe3 O4 /sSiO2 nanocomposites for pH-responsive MR imaging and oxygen-elevated synergetic therapy. Small.

[56] Chao Y, Wang G, Liang C, Yi X, Zhong X, Liu J, Gao M, Yang K, Cheng L, Liu Z (2016). Rhenium-188 labeled tungsten disulfide nanoflakes for self-sensitized, near-infrared enhanced radioisotope therapy. Small.

[57] Hao J, Song G, Liu T, Yi X, Yang K, Cheng L, Liu Z (2017). In vivo long-term biodistribution, excretion, and toxicology of pegylated transition-metal dichalcogenides MS2 (M = Mo, W, Ti) nanosheets. Adv Sci (Weinh).

[58] Liu T, Wang C, Gu X, Gong H, Cheng L, Shi X, Feng L, Sun B, Liu Z (2014). Drug delivery with PEGylated MoS2 nano-sheets for combined photothermal and chemotherapy of cancer. Adv Mater.

[59] Dong X, Yin W, Zhang X, Zhu S, He X, Yu J, Xie J, Guo Z, Yan L, Liu X (2018). Intelligent MoS2 nanotheranostic for targeted and Enzyme-/pH-/NIR-responsive drug delivery to overcome cancer chemotherapy resistance guided by PET imaging. ACS Appl Mater Interfaces.

[60] Kim J, Kim H, Kim WJ (2016). Single-layered MoS2-PEI-PEG nanocomposite-mediated gene delivery controlled by photo and redox stimuli. Small.

[61] Kim JE, Yim D, Han SW, Nam J, Kim JH, Kim JW (2019). Effective suppression of oxidative stress on living cells in hydrogel particles containing a physically immobilized WS2 radical scavenger. ACS Appl Mater Interfaces.

[62] Zhang A, Li A, Tian W, Li Z, Wei C, Sun Y, Zhao W, Liu M, Liu J (2017). A target-directed chemo-photothermal system based on transferrin and copolymer-modified MoS2 nanoplates with pH-activated drug release. Chemistry.

[63] Zhang Y, Zheng Y, Rui K, Hng HH, Hippalgaonkar K, Xu J, Sun W, Zhu J, Yan Q, Huang W (2017). 2D black phosphorus for energy storage and thermoelectric applications. Small.

[64] Tao W, Zhu X, Yu X, Zeng X, Xiao Q, Zhang X, Ji X, Wang X, Shi J, Zhang H, Mei L (2017). Black phosphorus nanosheets as a robust delivery platform for cancer theranostics. Adv Mater.

[65] Geng B, Shen W, Li P, Fang F, Qin H, Li XK, Pan D, Shen L (2019). Carbon dot-passivated black phosphorus nanosheet hybrids for synergistic cancer therapy in the NIR-II window. ACS Appl Mater Interfaces.

[66] Liu H, Neal AT, Zhu Z, Luo Z, Xu X, Tománek D, Ye PD (2014). Phosphorene: an unexplored 2D semiconductor with a high hole mobility. ACS Nano.

[67] Huang Y, Qiao J, He K, Bliznakov S, Sutter E, Chen X, Luo D, Meng F, Su D, Decker J (2016). Interaction of black phosphorus with oxygen and water. Chem Mater.

[68] Guo Z, Ding W, Liu X, Sun Z, Wei L (2019). Two-dimensional black phosphorus: a new star in energy applications and the barrier to stability. Appl Mater Today.

[69] Li Z, Guo T, Hu Y, Qiu Y, Liu Y, Wang H, Li Y, Chen X, Song J, Yang H (2019). A highly effective pi-pi stacking strategy to modify black phosphorus with aromatic molecules for cancer theranostics. ACS Appl Mater Interfaces.

[70] Zeng X, Luo M, Liu G, Wang X, Tao W, Lin Y, Ji X, Nie L, Mei L (2018). Polydopamine-modified black phosphorous nanocapsule with enhanced stability and photothermal performance for tumor multimodal treatments. Adv Sci (Weinh).

[71] Chen L, Chen C, Chen W, Li K, Chen X, Tang X, Xie G, Luo X, Wang X, Liang H, Yu S (2018). Biodegradable black phosphorus nanosheets mediate specific delivery of hTERT siRNA for synergistic cancer therapy. ACS Appl Mater Interfaces.

[72] Rives V, Del Arco M, Martin C (2013). Layered double hydroxides as drug carriers and for controlled release of non-steroidal antiinflammatory drugs (NSAIDs): a review. J Control Release.

[73] Xu M, Wei M (2018). Layered double hydroxide-based catalysts: recent advances in preparation, structure, and applications. Adv Func Mater.

[74] Kuthati Y, Kankala RK, Lee C-H (2015). Layered double hydroxide nanoparticles for biomedical applications: current status and recent prospects. Appl Clay Sci.

[75] Cao Z, Li B, Sun L, Li L, Xu ZP, Gu Z (2020). 2D layered double hydroxide nanoparticles: recent progress toward preclinical/clinical nanomedicine. Small Methods.

[76] Li B, Gu Z, Kurniawan N, Chen W, Xu ZP (2017). Manganese-based layered double hydroxide nanoparticles as a T1 -MRI contrast agent with ultrasensitive pH response and high relaxivity. Adv Mater.

[77] Mishra G, Dash B, Pandey S (2018). Layered double hydroxides: a brief review from fundamentals to application as evolving biomaterials. Appl Clay Sci.

[78] Deng H, Grunder S, Cordova KE, Valente C, Furukawa H, Hmadeh M, Gandara F, Whalley AC, Liu Z, Asahina S (2012). Large-pore apertures in a series of metal-organic frameworks. Science.

[79] Zhang X, Li G, Wu D, Zhang B, Hu N, Wang H, Liu J, Wu Y (2019). Recent advances in the construction of functionalized covalent organic frameworks and their applications to sensing. Biosens Bioelectron.

[80] Zheng W, Tsang C-S, Lee LYS, Wong K-Y (2019). Two-dimensional metal-organic framework and covalent-organic framework: synthesis and their energy-related applications. Mater Today Chem.

[81] Wang K, Zhang Z, Lin L, Chen J, Hao K, Tian H, Chen X (2019). Covalent organic nanosheets integrated heterojunction with two strategies to overcome hypoxic-tumor photodynamic therapy. Chem Mater.

[82] Li Y, Gao Z, Chen F, You C, Wu H, Sun K, An P, Cheng K, Sun C, Zhu X, Sun B (2018). Decoration of cisplatin on 2D metal-organic frameworks for enhanced anticancer effects through highly increased reactive oxygen species generation. ACS Appl Mater Interfaces.

[83] Wang Y, Zhao M, Ping J, Chen B, Cao X, Huang Y, Tan C, Ma Q, Wu S, Yu Y (2016). Bioinspired design of ultrathin 2D bimetallic metal-organic-framework nanosheets used as biomimetic enzymes. Adv Mater.

[84] Gan S, Tong X, Zhang Y, Wu J, Hu Y, Yuan A (2019). Covalent organic framework-supported molecularly dispersed near-infrared dyes boost immunogenic phototherapy against tumors. Adv Func Mater.

[85] Wang L, Guan S, Weng Y, Xu SM, Lu H, Meng X, Zhou S (2019). Highly efficient vacancy-driven photothermal therapy mediated by ultrathin MnO2 nanosheets. ACS Appl Mater Interfaces.

[86] Zhao Z, Fan H, Zhou G, Bai H, Liang H, Wang R, Zhang X, Tan W (2014). Activatable fluorescence/MRI bimodal platform for tumor cell imaging via MnO2 nanosheet-aptamer nanoprobe. J Am Chem Soc.

[87] Yao Y, Li N, Zhang X, Ong'achwa Machuki J, Yang D, Yu Y, Li J, Tang D, Tian J, Gao F (2019). DNA-Templated Silver Nanocluster/Porphyrin/MnO2 platform for label-free intracellular Zn(2+) imaging and fluorescence-/magnetic resonance imaging-guided photodynamic therapy. ACS Appl Mater Interfaces.

[88] Ma Z, Jia X, Bai J, Ruan Y, Wang C, Li J, Zhang M, Jiang X (2017). MnO2 gatekeeper: an intelligent and O2-evolving shell for preventing premature release of high cargo payload core, overcoming tumor hypoxia, and acidic H2O2-sensitive MRI. Adv Func Mater.

[89] Liu J, Chen Q, Zhu W, Yi X, Yang Y, Dong Z, Liu Z (2017). Nanoscale-coordination-polymer-shelled manganese dioxide composite nanoparticles: a multistage Redox/pH/H2O2-responsive cancer theranostic nanoplatform. Adv Func Mater.

[90] Chen Y, Ye D, Wu M, Chen H, Zhang L, Shi J, Wang L (2014). Break-up of two-dimensional MnO2 nanosheets promotes ultrasensitive pH-triggered theranostics of cancer. Adv Mater.

[91] Tang W, Fan W, Zhang W, Yang Z, Li L, Wang Z, Chiang YL, Liu Y, Deng L, He L (2019). Wet/Sono-chemical synthesis of enzymatic two-dimensional MnO2 nanosheets for synergistic catalysis-enhanced phototheranostics. Adv Mater.

[92] Chen Z, Wu C, Yuan Y, Xie Z, Li T, Huang H, Li S, Deng J, Lin H, Shi Z (2023). CRISPR-Cas13a-powered electrochemical biosensor for the detection of the L452R mutation in clinical samples of SARS-CoV-2 variants. J Nanobiotechnology.

[93] Zheng F, Chen Z, Li J, Wu R, Zhang B, Nie G, Xie Z, Zhang H (2022). A highly sensitive CRISPR-empowered surface plasmon resonance sensor for diagnosis of inherited diseases with femtomolar-level real-time quantification. Adv Sci (Weinh).

[94] Tian X, Yang Z, Duan G, Wu A, Gu Z, Zhang L, Chen C, Chai Z, Ge C, Zhou R (2017). Graphene oxide nanosheets retard cellular migration via disruption of actin cytoskeleton. Small.

[95] Wei S, Zou X, Tian J, Huang H, Guo W, Chen Z (2019). Control of protein conformation and orientation on graphene. J Am Chem Soc.

[96] Baimanov D, Wu J, Chu R, Cai R, Wang B, Cao M, Tao Y, Liu J, Guo M, Wang J (2020). Immunological responses induced by blood protein coronas on two-dimensional MoS(2) nanosheets. ACS Nano.

[97] Mo J, Xie Q, Wei W, Zhao J (2018). Revealing the immune perturbation of black phosphorus nanomaterials to macrophages by understanding the protein corona. Nat Commun.

[98] Tu Y, Lv M, Xiu P, Huynh T, Zhang M, Castelli M, Liu Z, Huang Q, Fan C, Fang H, Zhou R (2013). Destructive extraction of phospholipids from Escherichia coli membranes by graphene nanosheets. Nat Nanotechnol.

[99] Huang L, Zhang X, Ding Z, Qi Y, Wang W, Xu X, Yue H, Bai L, Wang H, Feng L (2022). PEGylated 2D-nanomaterials alleviate Parkinson's disease by shielding PIP2 lipids to inhibit IP3 second messenger signaling. Nano Today.

[100] Gu Z, Chen SH, Ding Z, Song W, Wei W, Liu S, Ma G, Zhou R (2019). The molecular mechanism of robust macrophage immune responses induced by PEGylated molybdenum disulfide. Nanoscale.

[101] Chen P, Yue H, Zhai X, Huang Z, Ma G-H, Wei W, Yan L-T (2019). Transport of a graphene nanosheet sandwiched inside cell membranes. Sci Adv.

[102] Zhang X, Ding Z, Ma G, Wei W (2021). A high-resolution ternary model demonstrates how PEGylated 2D nanomaterial stimulates integrin alpha(v) beta(8) on cell membrane. Adv Sci (Weinh).

[103] Zhang H, Luo B, An P, Zhan X, Lan F, Wu Y (2022). Interaction of nucleic acids with metal-organic framework nanosheets by fluorescence spectroscopy and molecular dynamics simulations. ACS Appl Bio Mater.

[104] Luo N, Weber JK, Wang S, Luan B, Yue H, Xi X, Du J, Yang Z, Wei W, Zhou R, Ma G (2017). PEGylated graphene oxide elicits strong immunological responses despite surface passivation. Nat Commun.

[105] Ding Z, Zhang X, Wang Y, Ogino K, Wu Y, Yue H, Jiao Z, Song C, Lu G, Wang S (2023). Nanomaterial's interfacial stimulation of vascular endothelial cells and divergent guidances for nanomedicine treating vasculature-associated diseases. Nano Today.

[106] Wang Q, Liu Y, Wang H, Jiang P, Qian W, You M, Han Y, Zeng X, Li J, Lu H (2022). Graphdiyne oxide nanosheets display selective anti-leukemia efficacy against DNMT3A-mutant AML cells. Nat Commun.

[107] Shao X, Ding Z, Zhou W, Li Y, Li Z, Cui H, Lin X, Cao G, Cheng B, Sun H (2021). Intrinsic bioactivity of black phosphorus nanomaterials on mitotic centrosome destabilization through suppression of PLK1 kinase. Nat Nanotechnol.

[108] Ji DK, Menard-Moyon C, Bianco A (2019). Physically-triggered nanosystems based on two-dimensional materials for cancer theranostics. Adv Drug Deliv Rev.

[109] Shim G, Kim MG, Park JY, Oh YK (2016). Graphene-based nanosheets for delivery of chemotherapeutics and biological drugs. Adv Drug Deliv Rev.

[110] Chen W, Ouyang J, Liu H, Chen M, Zeng K, Sheng J, Liu Z, Han Y, Wang L, Li J (2017). Black phosphorus nanosheet-based drug delivery system for synergistic photodynamic/photothermal/chemotherapy of cancer. Adv Mater.

[111] Zhang H, Zhang L, Cao Z, Cheong S, Boyer C, Wang Z, Yun SLJ, Amal R, Gu Z (2022). Two-dimensional ultra-thin nanosheets with extraordinarily high drug loading and long blood circulation for cancer therapy. Small.

[112] Nasrollahi F, Varshosaz J, Khodadadi AA, Lim S, Jahanian-Najafabadi A (2016). Targeted delivery of docetaxel by use of transferrin/poly(allylamine hydrochloride)-functionalized graphene oxide nanocarrier. ACS Appl Mater Interfaces.

[113] Pramanik N, Ranganathan S, Rao S, Suneet K, Jain S, Rangarajan A, Jhunjhunwala S (2019). A composite of hyaluronic acid-modified graphene oxide and iron oxide nanoparticles for targeted drug delivery and magnetothermal therapy. ACS Omega.

[114] Zhang L, Xia J, Zhao Q, Liu L, Zhang Z (2010). Functional graphene oxide as a nanocarrier for controlled loading and targeted delivery of mixed anticancer drugs. Small.

[115] Oz Y, Barras A, Sanyal R, Boukherroub R, Szunerits S, Sanyal A (2017). Functionalization of reduced graphene oxide via thiol-maleimide "click" chemistry: facile fabrication of targeted drug delivery vehicles. ACS Appl Mater Interfaces.

[116] Kim H, Lee D, Kim J, Kim T-I, Kim WJ (2013). Photothermally triggered cytosolic drug delivery via endosome disruption using a functionalized reduced graphene oxide. ACS Nano.

[117] Yin T, Liu J, Zhao Z, Zhao Y, Dong L, Yang M, Zhou J, Huo M (2017). Redox sensitive hyaluronic acid-decorated graphene oxide for photothermally controlled tumor-cytoplasm-selective rapid drug delivery. Adv Funct Mater.

[118] Wang Z, Cheng H, Sheng Y, Chen Z, Zhu X, Ren J, Zhang X, Lv L, Zhang H, Zhou J, Ding Y (2022). Biofunctionalized graphene oxide nanosheet for amplifying antitumor therapy: multimodal high drug encapsulation, prolonged hyperthermal window, and deep-site burst drug release. Biomaterials.

[119] Chen L, Qian M, Jiang H, Zhou Y, Du Y, Yang Y, Huo T, Huang R, Wang Y (2020). Multifunctional mesoporous black phosphorus-based nanosheet for enhanced tumor-targeted combined therapy with biodegradation-mediated metastasis inhibition. Biomaterials.

[120] Tao W, Ji X, Zhu X, Li L, Wang J, Zhang Y, Saw PE, Li W, Kong N, Islam MA (2018). Two-dimensional antimonene-based photonic nanomedicine for cancer theranostics. Adv Mater.

[121] Huang C, Sun Z, Cui H, Pan T, Geng S, Zhou W, Chu PK, Yu XF (2019). InSe nanosheets for efficient NIR-II-responsive drug release. ACS Appl Mater Interfaces.

[122] Liu Y, Ji X, Liu J, Tong WWL, Askhatova D, Shi J (2017). Tantalum sulfide nanosheets as a theranostic nanoplatform for computed tomography imaging-guided combinatorial chemo-photothermal therapy. Adv Funct Mater.

[123] Chen D, Jin Z, Zhao B, Wang Y, He Q (2021). MBene as a theranostic nanoplatform for photocontrolled intratumoral retention and drug release. Adv Mater.

[124] Ji X, Kong N, Wang J, Li W, Xiao Y, Gan ST, Zhang Y, Li Y, Song X, Xiong Q (2018). A novel top-down synthesis of ultrathin 2D boron nanosheets for multimodal imaging-guided cancer therapy. Adv Mater.

[125] Wang F, Duan H, Zhang R, Guo H, Lin H, Chen Y (2020). Potentiated cytosolic drug delivery and photonic hyperthermia by 2D free-standing silicene nanosheets for tumor nanomedicine. Nanoscale.

[126] Feng C, Ouyang J, Tang Z, Kong N, Liu Y, Fu L, Ji X, Xie T, Farokhzad OC, Tao W (2020). Germanene-based theranostic materials for surgical adjuvant treatment: inhibiting tumor recurrence and wound infection. Matter.

[127] Xu L, Xue Y, Xia J, Qu X, Lei B, Yang T, Zhang X, Li N, Zhao H, Wang M (2020). Construction of high quality ultrathin lanthanide oxyiodide nanosheets for enhanced CT imaging and anticancer drug delivery to efficient cancer theranostics. Biomaterials.

[128] Xu Z, Zhu S, Wang M, Li Y, Shi P, Huang X (2015). Delivery of paclitaxel using PEGylated graphene oxide as a nanocarrier. ACS Appl Mater Interfaces.

[129] Li Y, Xiong J, Guo W, Jin Y, Miao W, Wang C, Zhang H, Hu Y, Huang H (2021). Decomposable black phosphorus nano-assembly for controlled delivery of cisplatin and inhibition of breast cancer metastasis. J Control Release.

[130] Huang D, Wang G, Mao J, Liu C, Fan Z, Zhang Y, Zhang B, Zhao Y, Dai C, He Y (2021). Intravital whole-process monitoring thermo-chemotherapy via 2D silicon nanoplatform: a macro guidance and long-term microscopic precise imaging strategy. Adv Sci (Weinh).

[131] Li L, Gu W, Chen J, Chen W, Xu ZP (2014). Co-delivery of siRNAs and anti-cancer drugs using layered double hydroxide nanoparticles. Biomaterials.

[132] Wang X, Cheng Y, Han X, Yan J, Wu Y, Song P, Wang Y, Li X, Zhang H (2022). Functional 2D iron-based nanosheets for synergistic immunotherapy, phototherapy, and chemotherapy of tumor. Adv Healthc Mater.

[133] Truong Hoang Q, Huynh KA, Nguyen Cao TG, Kang JH, Dang XN, Ravichandran V, Kang HC, Lee M, Kim JE, Ko YT (2023). Piezocatalytic 2D WS(2) nanosheets for ultrasound-triggered and mitochondria-targeted piezodynamic cancer therapy synergized with energy metabolism-targeted chemotherapy. Adv Mater.

[134] Ji Q, Yamazaki T, Sun J, Gorecka Z, Huang NC, Hsu SH, Shrestha LK, Hill JP, Ariga K (2017). Spongelike porous silica nanosheets: from "soft" molecular trapping to DNA delivery. ACS Appl Mater Interfaces.

[135] Senapati S, Sarkar T, Das P, Maiti P (2019). Layered double hydroxide nanoparticles for efficient gene delivery for cancer treatment. Bioconjug Chem.

[136] Chen S, Yang J, Liang Z, Li Z, Xiong W, Fan Q, Shen Z, Liu J, Xu Y (2023). Synergistic functional nanomedicine enhances ferroptosis therapy for breast tumors by a blocking defensive redox system. ACS Appl Mater Interfaces.

[137] Zhang Y, Teng Z, Ni Q, Tao J, Cao X, Wen Y, Wu L, Fang C, Wan B, Zhang X, Lu G (2020). Orderly curled silica nanosheets with a small size and macromolecular loading pores: synthesis and delivery of macromolecules to eradicate drug-resistant cancer. ACS Appl Mater Interfaces.

[138] Liu L, Du X (2021). Polyethylenimine-modified graphitic carbon nitride nanosheets: a label-free Raman traceable siRNA delivery system. J Mater Chem B.

[139] Luan X, Guan YY, Liu HJ, Lu Q, Zhao M, Sun D, Lovell JF, Sun P, Chen HZ, Fang C (2018). A tumor vascular-targeted interlocking trimodal nanosystem that induces and exploits hypoxia. Adv Sci (Weinh).

[140] Teimouri M, Nia AH, Abnous K, Eshghi H, Ramezani M (2016). Graphene oxide-cationic polymer conjugates: synthesis and application as gene delivery vectors. Plasmid.

[141] Yadav N, Kumar N, Prasad P, Shirbhate S, Sehrawat S, Lochab B (2018). stable dispersions of covalently tethered polymer improved graphene oxide nanoconjugates as an effective vector for siRNA delivery. ACS Appl Mater Interfaces.

[142] Li H, Liu M, Zhang S, Xie X, Zhu Y, Liu T, Li J, Tu Z, Wen W (2023). Construction of CpG delivery nanoplatforms by functionalized MoS(2) nanosheets for boosting antitumor immunity in head and neck squamous cell carcinoma. Small.

[143] Li WH, Wu JJ, Wu L, Zhang BD, Hu HG, Zhao L, Li ZB, Yu XF, Li YM (2021). Black phosphorous nanosheet: a novel immune-potentiating nanoadjuvant for near-infrared-improved immunotherapy. Biomaterials.

[144] Song C, Li F, Wang S, Wang J, Wei W, Ma G (2019). Recent advances in particulate adjuvants for cancer vaccination. Adv Ther.

[145] Chen Y, De Koker S, De Geest BG (2020). Engineering strategies for lymph node targeted immune activation. Acc Chem Res.

[146] Zhu C, Jiang J, Jia Y, Xu ZP, Zhang L (2023). Beyond drug delivery system: immunomodulatory layered double hydroxide nanoadjuvants take an essential step forward in cancer immunotherapy. Acc Mater Res.

[147] Zhang LX, Xie XX, Liu DQ, Xu ZP, Liu RT (2018). Efficient co-delivery of neo-epitopes using dispersion-stable layered double hydroxide nanoparticles for enhanced melanoma immunotherapy. Biomaterials.

[148] Yue H, Wei W, Gu Z, Ni D, Luo N, Yang Z, Zhao L, Garate JA, Zhou R, Su Z, Ma G (2015). Exploration of graphene oxide as an intelligent platform for cancer vaccines. Nanoscale.

[149] Xu C, Hong H, Lee Y, Park KS, Sun M, Wang T, Aikins ME, Xu Y, Moon JJ (2020). Efficient lymph node-targeted delivery of personalized cancer vaccines with reactive oxygen species-inducing reduced graphene oxide nanosheets. ACS Nano.

[150] Tang F, Wang Y, Zeng Y, Xiao A, Tong A, Xu J (2023). Tumor-associated macrophage-related strategies for glioma immunotherapy. NPJ Precis Oncol.

[151] Guo M, Zhao L, Liu J, Wang X, Yao H, Chang X, Liu Y, Liu J, You M, Ren J (2021). The underlying function and structural organization of the intracellular protein corona on graphdiyne oxide nanosheet for local immunomodulation. Nano Lett.

[152] Guo M, Liu J, Chen X, You Z, Gao F, Liu T, Ren J, Liu J, Xiong Z, Liu Y (2022). Graphdiyne oxide nanosheets reprogram immunosuppressive macrophage for cancer immunotherapy. Nano Today.

[153] Liang C, Xiong N, Liu M, Chen Y, Li W, Xu J, Sun Y, Wang Y, Dong Y, Fan W (2023). Manganese immunotherapy for treating osteosarcoma: glycosylating 1V209 anchored MnO2 nanosheets prompt pro-inflammatory macrophage polarization. Nano Today.

[154] He C, Yu L, Ding L, Yao H, Chen Y, Hao Y (2020). Lysine demethylase KDM3A regulates nanophotonic hyperthermia resistance generated by 2D silicene in breast cancer. Biomaterials.

[155] Lin H, Qiu W, Liu J, Yu L, Gao S, Yao H, Chen Y, Shi J (2019). Silicene: wet-chemical exfoliation synthesis and biodegradable tumor nanomedicine. Adv Mater.

[156] Hu R, Dai C, Wang C, Lin J, Hu H, Li Z, Lin H, Ding L, Chen Y, Zhang B (2021). Engineering 2D arsenic-phosphorus theranostic nanosheets. Adv Funct Mater.

[157] Gao G, Jiang YW, Jia HR, Sun W, Guo Y, Yu XW, Liu X, Wu FG (2019). From perinuclear to intranuclear localization: a cell-penetrating peptide modification strategy to modulate cancer cell migration under mild laser irradiation and improve photothermal therapeutic performance. Biomaterials.

[158] Liu Q, Xie Z, Qiu M, Shim I, Yang Y, Xie S, Yang Q, Wang D, Chen S, Fan T (2020). Prodrug-loaded zirconium carbide nanosheets as a novel biophotonic nanoplatform for effective treatment of cancer. Adv Sci (Weinh).

[159] Qiu M, Wang D, Liang W, Liu L, Zhang Y, Chen X, Sang DK, Xing C, Li Z, Dong B (2018). Novel concept of the smart NIR-light-controlled drug release of black phosphorus nanostructure for cancer therapy. Proc Natl Acad Sci U S A.

[160] Zhu X, Zhang W, Xiang H, Chang Q, Liu R, Wan Y, Zhang R, Zhao F, She Y, Yuan H (2023). Radiation-enhanced self-cascade catalytic Ti3C2Tx-based platform enables controlled release of trans-resveratrol for synergistic radiosensitization against metastasis of orthotopic breast cancer. Nano Today.

[161] Zhu Y, Wang Z, Zhao R, Zhou Y, Feng L, Gai S, Yang P (2022). Pt decorated Ti(3)C(2)T(x) MXene with NIR-II light amplified nanozyme catalytic activity for efficient phototheranostics. ACS Nano.

[162] Zhu Y, Tang X, Liu Q, Xia Y, Zhai X, Zhang H, Duan D, Wang H, Zhan W, Wu L (2022). Metallic carbonitride mxene based photonic hyperthermia for tumor therapy. Small.

[163] Xiang H, Lin H, Yu L, Chen Y (2019). Hypoxia-irrelevant photonic thermodynamic cancer nanomedicine. ACS Nano.

[164] Zeng K, Xu Q, Ouyang J, Han Y, Sheng J, Wen M, Chen W, Liu YN (2019). Coordination nanosheets of phthalocyanine as multifunctional platform for imaging-guided synergistic therapy of cancer. ACS Appl Mater Interfaces.

[165] Liu Z, Zhang S, Lin H, Zhao M, Yao H, Zhang L, Peng W, Chen Y (2018). Theranostic 2D ultrathin MnO(2) nanosheets with fast responsibility to endogenous tumor microenvironment and exogenous NIR irradiation. Biomaterials.

[166] Dai C, Zhang S, Liu Z, Wu R, Chen Y (2017). Two-dimensional graphene augments nanosonosensitized sonocatalytic tumor eradication. ACS Nano.

[167] Chen S, Xing C, Huang D, Zhou C, Ding B, Guo Z, Peng Z, Wang D, Zhu X, Liu S (2020). Eradication of tumor growth by delivering novel photothermal selenium-coated tellurium nanoheterojunctions. Sci Adv.

[168] Lian Y, Wang C, Meng Y, Dong J, Zhang J, Xu S, Bai G, Gao J (2023). Selenide heterostructure nanosheets with efficient near-infrared photothermal conversion for therapy. ACS Omega.

[169] Hui T, Fu J, Zheng B, Fu C, Zhao B, Zhang T, Zhang Y, Wang C, Yu L, Yang Y (2023). Subtractive nanopore engineered mxene photonic nanomedicine with enhanced capability of photothermia and drug delivery for synergistic treatment of osteosarcoma. ACS Appl Mater Interfaces.

[170] Hu T, Yan L, Wang Z, Shen W, Liang R, Yan D, Wei M (2021). A pH-responsive ultrathin Cu-based nanoplatform for specific photothermal and chemodynamic synergistic therapy. Chem Sci.

[171] Liu J, Smith S, Wang C (2022). Reversing the epithelial-mesenchymal transition in metastatic cancer cells using CD146-targeted black phosphorus nanosheets and a mild photothermal treatment. ACS Nano.

[172] Liu J, Smith S, Wang C (2023). Photothermal attenuation of cancer cell stemness, chemoresistance, and migration using CD44-targeted MoS(2) nanosheets. Nano Lett.

[173] Seung Lee J, Kim J, Ye YS, Kim TI (2022). Materials and device design for advanced phototherapy systems. Adv Drug Deliv Rev.

[174] Zhu W, Yang Y, Jin Q, Chao Y, Tian L, Liu J, Dong Z, Liu Z (2019). Two-dimensional metal-organic-framework as a unique theranostic nano-platform for nuclear imaging and chemo-photodynamic cancer therapy. Nano Res.

[175] Liu M, Zhu H, Wang Y, Sevencan C, Li BL (2021). Functionalized MoS2-based nanomaterials for cancer phototherapy and other biomedical applications. ACS Mater Lett.

[176] Nie Y, Zhang W, Xiao W, Zeng W, Chen T, Huang W, Wu X, Kang Y, Dong J, Luo W, Ji X (2022). Novel biodegradable two-dimensional vanadene augmented photoelectro-fenton process for cancer catalytic therapy. Biomaterials.

[177] Shen W, Hu T, Liu X, Zha J, Meng F, Wu Z, Cui Z, Yang Y, Li H, Zhang Q (2022). Defect engineering of layered double hydroxide nanosheets as inorganic photosensitizers for NIR-III photodynamic cancer therapy. Nat Commun.

[178] Zhang Y, Jia Q, Li J, Wang J, Liang K, Xue X, Chen T, Kong L, Ren H, Liu W (2023). Copper-bacteriochlorin nanosheet as a specific pyroptosis inducer for robust tumor immunotherapy. Adv Mater.

[179] Xu D, Lin H, Qiu W, Ge M, Chen Z, Wu C, You Y, Lu X, Wei C, Liu J (2021). Hydrogen-bonded silicene nanosheets of engineered bandgap and selective degradability for photodynamic therapy. Biomaterials.

[180] Yue H, Wei W, Yue Z, Wang B, Luo N, Gao Y, Ma D, Ma G, Su Z (2012). The role of the lateral dimension of graphene oxide in the regulation of cellular responses. Biomaterials.

[181] Wang Y, Li M, Wang S, Ma J, Liu Y, Guo H, Gao J, Yao L, He B, Hu L (2022). Deciphering the effects of 2D black phosphorus on disrupted hematopoiesis and pulmonary immune homeostasis using a developed flow cytometry method. Environ Sci Technol.

[182] Li J, Wang X, Mei KC, Chang CH, Jiang J, Liu X, Liu Q, Guiney LM, Hersam MC, Liao YP (2021). Lateral size of graphene oxide determines differential cellular uptake and cell death pathways in Kupffer cells, LSECs, and hepatocytes. Nano Today.

[183] Luo N, Ni D, Yue H, Wei W, Ma G (2015). Surface-engineered graphene navigate divergent biological outcomes toward macrophages. ACS Appl Mater Interfaces.

[184] Li J, Guiney LM, Downing JR, Wang X, Chang CH, Jiang J, Liu Q, Liu X, Mei KC, Liao YP (2021). Dissolution of 2D molybdenum disulfide generates differential toxicity among liver cell types compared to non-toxic 2D boron nitride effects. Small.

[185] Ding Z, Luo N, Yue H, Gao Y, Ma G, Wei W (2020). In vivo immunological response of exposure to PEGylated graphene oxide via intraperitoneal injection. J Mater Chem B.

[186] Lin Y, Zhang Y, Li J, Kong H, Yan Q, Zhang J, Li W, Ren N, Cui Y, Zhang T (2020). Blood exposure to graphene oxide may cause anaphylactic death in non-human primates. Nano Today.

[187] Kozma GT, Shimizu T, Ishida T, Szebeni J (2020). Anti-PEG antibodies: properties, formation, testing and role in adverse immune reactions to PEGylated nano-biopharmaceuticals. Adv Drug Deliv Rev.

[188] Fan T, Yan L, He S, Hong Q, Ai F, He S, Ji T, Hu X, Ha E, Zhang B (2022). Biodistribution, degradability and clearance of 2D materials for their biomedical applications. Chem Soc Rev.

[189] Visani de Luna LA, Loret T, He Y, Legnani M, Lin H, Galibert AM, Fordham A, Holme S, Del Rio Castillo AE, Bonaccorso F (2023). Pulmonary toxicity of boron nitride nanomaterials is aspect ratio dependent. ACS Nano.

[190] Cao M, Cai R, Zhao L, Guo M, Wang L, Wang Y, Zhang L, Wang X, Yao H, Xie C (2021). Molybdenum derived from nanomaterials incorporates into molybdenum enzymes and affects their activities in vivo. Nat Nanotechnol.

[191] Im HJ, England CG, Feng L, Graves SA, Hernandez R, Nickles RJ, Liu Z, Lee DS, Cho SY, Cai W (2016). accelerated blood clearance phenomenon reduces the passive targeting of PEGylated nanoparticles in peripheral arterial disease. ACS Appl Mater Interfaces.

